# Genetic studies of human–chimpanzee divergence using stem cell fusions

**DOI:** 10.1073/pnas.2117557118

**Published:** 2021-12-17

**Authors:** Janet H. T. Song, Rachel L. Grant, Veronica C. Behrens, Marek Kučka, Garrett A. Roberts Kingman, Volker Soltys, Yingguang Frank Chan, David M. Kingsley

**Affiliations:** ^a^Department of Developmental Biology, Stanford University School of Medicine, Stanford, CA 94305;; ^b^Department of Genetics, Stanford University School of Medicine, Stanford, CA 94305;; ^c^Friedrich Miescher Laboratory of the Max Planck Society, 72076 Tübingen, Germany;; ^d^HHMI, Stanford University School of Medicine, Stanford, CA 94305

**Keywords:** human–chimpanzee evolution, tetraploid, *cis*/*trans* gene regulation, genetic mapping

## Abstract

Complete genome sequencing has identified millions of DNA changes that differ between humans and chimpanzees. Although a subset of these changes likely underlies important phenotypic differences between humans and chimpanzees, it is currently difficult to distinguish causal from incidental changes and to map specific phenotypes to particular genome locations. To facilitate further genetic study of human–chimpanzee divergence, we have generated human and chimpanzee autotetraploids and allotetraploids by fusing induced pluripotent stem cells (iPSCs) of each species. The resulting tetraploid iPSCs can be stably maintained and retain the ability to differentiate along ectoderm, mesoderm, and endoderm lineages. RNA sequencing identifies thousands of genes whose expression differs between humans and chimpanzees when assessed in single-species diploid or autotetraploid iPSCs. Analysis of gene expression patterns in interspecific allotetraploid iPSCs shows that human–chimpanzee expression differences arise from substantial contributions of both *cis*-acting changes linked to the genes themselves and *trans*-acting changes elsewhere in the genome. To enable further genetic mapping of species differences, we tested chemical treatments for stimulating genome-wide mitotic recombination between human and chimpanzee chromosomes, and CRISPR methods for inducing species-specific changes on particular chromosomes in allotetraploid cells. We successfully generated derivative cells with nested deletions or interspecific recombination on the X chromosome. These studies confirm an important role for the X chromosome in *trans* regulation of expression differences between species and illustrate the potential of this system for more detailed *cis* and *trans* mapping of the molecular basis of human and chimpanzee evolution.

## Significance

Comparative studies of humans and chimpanzees have revealed many anatomical, physiological, behavioral, and molecular differences. However, it has been challenging to map these differences to particular chromosome regions. Here, we develop a genetic approach in fused stem cell lines that makes it possible to map human–chimpanzee molecular and cellular differences to specific regions of the genome. We illustrate this approach by mapping chromosome regions responsible for species-specific gene expression differences in fused tetraploid cells. This approach is general, and could be used in the future to map the genomic changes that control many other human–chimpanzee differences in various cell types or organoids in vitro.

Humans have had a long-standing interest in the features that distinguish our species from other animals (1, 2). Comparative studies have characterized many morphological, physiological, and behavioral similarities and differences among great apes (3). Paleontological studies have traced the origin and timing of the appearance of various human features in the fossil record (4). More recently, advances in sequencing technologies have allowed for the comparative genomic analysis of humans, chimpanzees, other nonhuman primates, and even extinct archaic human lineages such as Neanderthals and Denisovans (5).

Whole-genome comparisons indicate that ∼4% of the base pairs in the human genome differ from those in chimpanzees. Sifting through this set of ∼125 million DNA changes to separate the causal mutations contributing to phenotypic differences between humans and chimpanzees from inconsequential or neutral changes is a daunting problem, and has been compared to searching for needles in a haystack (3).

In evolutionary studies of other organisms, genetic crosses between different lineages have helped localize and prioritize chromosome regions that influence different traits. The formation of F1 hybrids, followed by chromosome recombination during meiosis, can be used to produce F2 offspring that inherit different combinations of alleles from the parental lineages. By comparing different genotypes and phenotypes across a large panel of meiotic mapping progeny, it has now been possible to map some evolutionary traits to particular chromosome regions in yeast, fruit flies, butterflies, sticklebacks, mice, and other organisms (6).

Traditional meiotic mapping approaches are limited to organisms that can be crossed to produce viable and fertile offspring. However, related approaches have also been developed for comparing genotypes and phenotypes in somatic cells without meiosis, when traditional crosses are not possible. Cells of even distantly related organisms can be fused in vitro to produce somatic cell hybrids that contain the genetic information from both lineages. The fused cells sometimes lose chromosomes of one or the other starting species, producing progeny cell lines that can be used to assign genes or cellular phenotypes to particular chromosomes (7). Hybrids can also be irradiated to fragment chromosomes and stimulate additional segregation of genetic information, an approach that has been used for fine mapping of genomic linkage relationships (8). Mitotic recombination within cultured cells can also be stimulated by mutations in DNA pathways, by chemicals that damage DNA, or by targeted breaks induced by Cas9 and guide RNAs (gRNAs) designed to alter particular locations in the genome. Mutations and chemical inhibitors of the Bloom Syndrome helicase gene (*BLM*) have been used to recover homozygous mutants in somatic cell gene screens (9, 10) or to induce recombination between chromosomes of distantly related mouse strains for studies of the genomic basis of evolutionary differences (11). The ability to induce breaks at particular loci with CRISPR-Cas9 has also made it possible to choose both the location and the direction of recombination between genomes in nonmeiotic cells, enabling high-resolution mapping without traditional crosses in yeast (12).

Development of similar approaches for human and chimpanzee cells would be very useful for studying the genomic basis of evolutionary differences that have evolved in hominids. Many molecular and cellular phenotypes that can be assayed and scored under cell culture conditions are known to differ between humans and chimpanzees. Recent studies have generated well-matched sets of human and chimpanzee induced pluripotent stem cell (iPSC) lines (13), and have shown that human and chimpanzee iPSCs can be fused to produce hybrids useful for comparing species-specific expression in cortical spheroids and neural crest cells (14, 15). Here we generate both autotetraploid (same species) and allotetraploid (different species) fusion lines from human and chimpanzee iPSCs, and use them to identify whether gene expression differences are due to *cis*- or *trans*-acting differences between species. We also test both random and targeted methods for stimulating DNA breaks and chromosome exchanges in allotetraploid iPSCs, providing a general method for further localizing the specific genomic changes that underlie human and chimpanzee differences in vitro.

## Results

### Generation and Initial Characterization of Autotetraploid and Allotetraploid iPSC Lines.

**Fig. 1.**
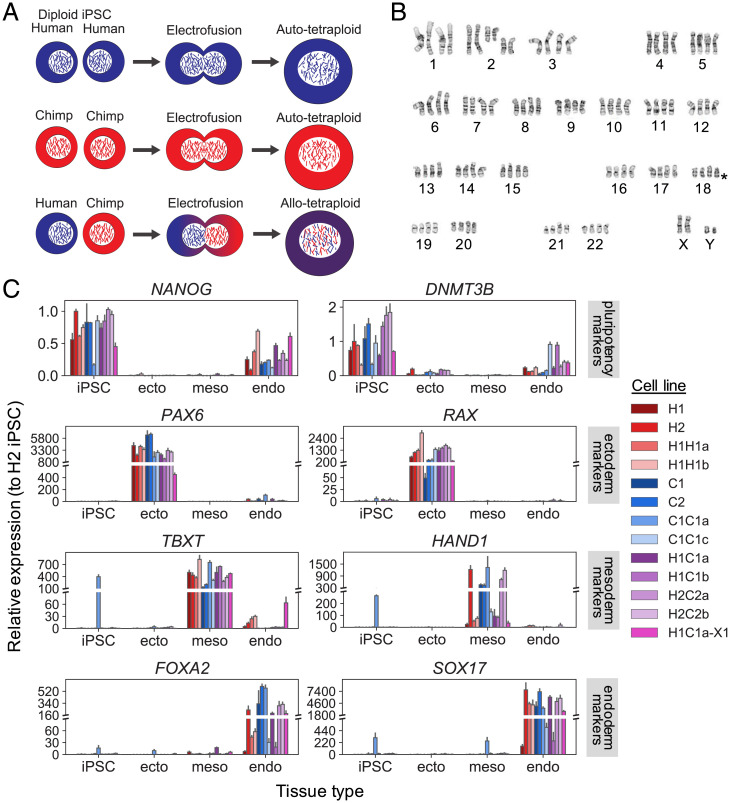
Generation and differentiation of autotetraploid and allotetraploid iPSC lines. Autotetraploid and allotetraploid cells contain the expected number of chromosomes and express expected marker genes after trilineage differentiation. (*A*) Human and chimpanzee diploid iPSC lines were labeled with diffusible dyes and subjected to electrofusion to generate autotetraploid and allotetraploid iPSC lines. (*B*) Tetraploid lines (H2C2a shown) exhibit karyotypes with four copies of each chromosome. Asterisk denotes location of a common iPSC human chr18q deletion (16), present in a subset of our cell lines. See Dataset S1 for detailed karyotype description of all lines. (*C*) Relative expression of pluripotency (*NANOG*, *DNMT3B*), ectoderm (*PAX6*, *RAX*), mesoderm (*TBXT*, *HAND1*), and endoderm (*FOXA2*, *SOX17*) marker genes tested via qRT-PCR after incubating cell lines under trilineage differentiation conditions. Cell lines tested are two human diploid lines (H1, H2), two human autotetraploid lines (H1H1a, H1H1b), two chimpanzee diploid lines (C1, C2), two chimpanzee autotetraploid lines (C1C1a, C1C1c), four allotetraploid lines (H1C1a, H1C1b, H2C2a, H2C2b), and one fluorescently marked allotetraploid line (H1C1a-X1). Gene expression is plotted relative to a human diploid undifferentiated iPSC line (H2). Error bars represent the SD of *N* = 3 cell culture replicates maintained as iPSCs or differentiated independently; 146 of 156 gene expression differences between undifferentiated cells and the tissue type in which a marker is expected to be expressed are significant by two-tailed Student’s *t* test at 5% FDR (see Dataset S3 for complete *P* value list).

To generate autotetraploids and allotetraploids, we labeled human and chimpanzee iPSC lines (13) with diffusible fluorescent dyes and fused them using electrofusion (Fig. 1*A* and *Materials and Methods*). Tetraploid cells were enriched by either fluorescence-activated cell sorting (FACS) or manual inspection and grown clonally. Successful fusion in expanded clones was confirmed by FACS analysis for DNA content using propidium iodide and by karyotyping. In total, we generated two human autotetraploid lines (“H1H1” lines, from human iPSC line H23555 [H1]); five chimpanzee autotetraploid lines (“C1C1” lines from chimpanzee iPSC line C3649 [C1]); and 22 human–chimpanzee allotetraploid lines from different fusion events including 12 “H1C1” lines derived from H1 and C1 and 10 “H2C2” lines derived from human iPSC line H20961 (H2) and chimpanzee iPSC line C8861 (C2) (Dataset S1).

Tetraploid iPSCs were larger than diploid cells but had normal morphology and could be routinely propagated under the same conditions as diploid iPSCs (*SI Appendix*, Fig. S1). We performed G-banded karyotyping on the initial diploid parental lines, as well as the newly generated autotetraploid and allotetraploid lines to examine their genome stability (Dataset S1). Fusion lines showed the tetraploid karyotypes expected from fusing their originating diploid lines. However, some of the tetraploid lines contained additional chromosomal abnormalities, including aneuploidies common to diploid human iPSC cultures (16) such as deletion of human chr18q (asterisk in Fig. 1*B*).

To assess the pluripotency and differentiation potential of the tetraploid iPSC lines, we differentiated representative diploid (H1, H2, C1, C2), autotetraploid (H1H1a, H1H1b, C1C1a, C1C1c), and allotetraploid (H1C1a, H1C1b, H2C2a, H2C2b) lines into ectoderm, mesoderm, and endoderm (*Materials and Methods*). Quantitative PCR (qPCR) for the expression of pluripotency (*NANOG*, *DNMT3B*), ectoderm (*PAX6*, *RAX*), mesoderm (*TBXT*, *HAND1*), and endoderm (*FOXA2*, *SOX17*) markers showed specific differentiation of tetraploid lines into all three lineages (Fig. 1*C*, Dataset S3, and *SI Appendix*, *Supplemental Materials and Methods*). For endoderm differentiation, a subset of lines (H1, C1C1c, H1C1b) showed lower expression of endoderm markers compared to all other cell lines, as well as persistent expression of pluripotency marker genes. Tetraploid cells thus retain broad differentiation abilities, but conditions may need to be optimized for particular cell lines or differentiation endpoints.

### Diploid and Autotetraploid iPSC Lines Have Similar Gene Expression Profiles.

To examine whether tetraploidization altered normal gene expression patterns, we used RNA sequencing (RNAseq) to characterize transcriptional differences due to ploidy, but not to species differences (i.e., H1 vs. H1H1 and C1 vs. C1C1). At a false discovery rate (FDR) of 5%, we detected 189 differentially expressed genes between H1 and H1H1, and 181 differentially expressed genes between C1 and C1C1, with at least a twofold change in expression (Dataset S4 and *SI Appendix*, *Supplemental Materials and Methods*). Neither set of differentially expressed genes was enriched for gene ontology categories (*SI Appendix*, *Supplemental Materials and Methods*), and only 13 genes were differentially expressed in both H1 compared to H1H1 and C1 compared to C1C1. We conclude that the creation of tetraploid cells alone does not activate a coordinated set of gene expression changes.

**Fig. 2.**
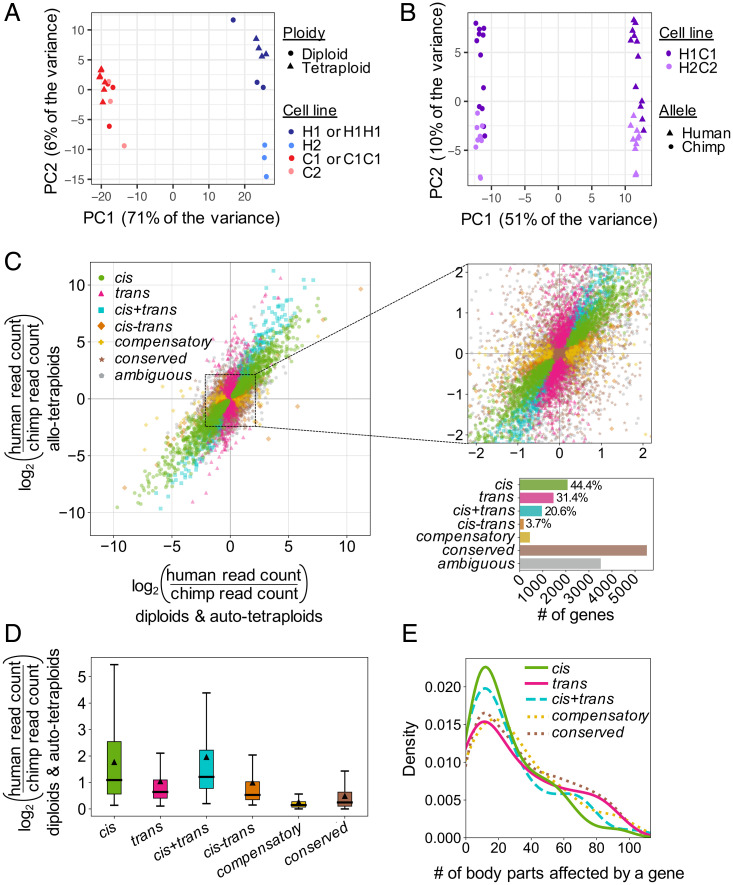
Gene expression profiling of human and chimpanzee diploid, autotetraploid, and allotetraploid iPSC lines. Tetraploidization does not result in coordinated gene expression changes, but thousands of genes are expressed differently between human and chimpanzee iPSCs due to a mixture of *cis*- and *trans*-regulatory changes. (*A*) Principal component analysis (PCA) of RNAseq of H1, H2, C1, C2, H1H1, and C1C1 diploid and autotetraploid iPSC lines. The cell lines cluster by species along PC1 and by cell line along PC2. Autotetraploid lines cluster with their cognate diploid line. (*B*) PCA of RNAseq of H1C1 and H2C2 allotetraploid lines. Allotetraploid lines are each represented by two dots, one for reads mapping to the human transcriptome and one for reads mapping to the chimpanzee transcriptome (*Materials and Methods*). Expression from human alleles (triangles) cluster separately from chimpanzee alleles (circles) in allotetraploid lines along PC1. PC2 separates the two sets of allotetraploid cell lines. (*C*) Each gene’s expression pattern was classified by regulatory type (*cis*, *trans*, *cis*+*trans*, *cis*–*trans*, compensatory, conserved, or ambiguous) by comparing DE between human- and chimpanzee-only iPSCs (*x* axis) and allele-specific gene expression between human and chimpanzee alleles within allotetraploid iPSCs (*y* axis). (*Left*) Data for all genes. (*Upper Right*) Zoom-in of dense center region. (*Lower Right*) Bar graph indicating number of genes per category and relative contribution (percentage) of each category to genes with human–chimpanzee regulatory differences. (*D*) Box plot showing distribution of effect sizes for gene expression changes in each regulatory category. Median effect size is indicated by thick horizontal lines, and mean effect size is indicated by triangles. All pairwise comparisons are statistically significant (adjusted *P* < 0.012 by two-tailed Mann–Whitney *U* test). (*E*) Density plot (smoothed histogram) showing the distribution of body parts influenced by genes [according to the Gene ORGANizer database (20)] in each regulatory category. For genes classified as *cis*, *trans*, and *cis*+*trans*, only genes with $|log_{2}(FC)|\geq 1$ are plotted. The *cis*–*trans* category is not included because only five genes have $|log_{2}(FC)|\geq 1$. Note that genes classified as *cis* or *cis*+*trans* tend to influence fewer body parts than conserved genes (median 18 body parts for both *cis* and *cis*+*trans* genes compared to median 30 body parts for conserved genes, adjusted *P* = 0.00028 and *P* = 0.0035 by two-tailed Mann–Whitney *U* test after FDR correction). This trend is not observed for *trans* and compensatory regulatory types (median 24 and 27 body parts, adjusted *P* = 0.11 and *P* = 0.21, respectively).

To assess gene expression variability between different cell lines from the same species, we also profiled global RNA patterns from a second set of human and chimpanzee diploid iPSC lines. We detected 410 differentially expressed genes between H1 and H2 and 181 differentially expressed genes between C1 and C2 at an FDR of 5% with at least a twofold change in expression (Dataset S4). Using principal component analysis, we found that global transcriptional profiles grouped by species, with human-derived lines clustering separately from chimpanzee-derived lines, and that diploid lines clustered more closely with their derived autotetraploid line than another diploid line of the same species (Fig. 2*A*). These results indicated that the transcriptional profiles of the diploid lines and their derived autotetraploid lines were at least as similar as the transcriptional profiles of two diploid lines from the same species. Taken together, our data suggest that tetraploid iPSCs behave similarly to diploid iPSCs at the level of gene expression.

### Differential Gene Expression and Allele-Specific Gene Expression Reveal Human- and Chimpanzee-Specific Gene Expression Profiles.

We next used our RNAseq data to identify gene expression differences between human and chimpanzee iPSCs (Dataset S5). Differential gene expression (DE) analysis between human-only and chimpanzee-only iPSC lines identified 5,984 genes differentially expressed between species. There were no significant gene ontology enrichments for DE genes with at least a twofold change in expression (*SI Appendix*, *Supplemental Materials and Methods*). Allele-specific expression (ASE) comparisons between the human allele and the chimpanzee allele in allotetraploid iPSC lines identified 4,540 allele-specific expressed genes. ASE results from this study and the ASE results from a previous study (14) that independently generated human–chimpanzee allotetraploid fusions from similar diploid iPSC lines were highly concordant (Pearson’s *r* = 0.72; *SI Appendix*, Fig. S2), suggesting that human–chimpanzee gene expression differences are robust and reproducible across laboratories.

### *cis*- and *trans*-Acting Regulatory Changes Are Both Important Contributors to Human–Chimpanzee Gene Expression Differences.

Determining whether gene expression differences between two species are due to *cis*-acting or *trans*-acting regulatory changes is possible when gene expression can be compared between each single species and a hybrid (17). We therefore leveraged the RNAseq data from human-only, chimpanzee-only, and human–chimpanzee allotetraploid iPSC lines to determine the regulatory type for genes that were differentially expressed between human-only and chimpanzee-only iPSCs (*Materials and Methods*). Specifically, when a *cis*-acting regulatory change causes a gene to be differentially expressed, the expression difference should be maintained in allotetraploid cells where both human and chimpanzee alleles are in the same *trans*-acting environment. Conversely, when a *trans*-acting regulatory change causes a gene to be differentially expressed, the expression difference should disappear in allotetraploid cell lines.

Our regulatory type classifications identified 5,956 genes with no net regulatory changes between our human-only and chimpanzee-only iPSC lines. Of these, 92.6% (5,515 genes) were classified as conserved between human and chimpanzee, and 7.4% (441 genes) were classified as compensatory (*cis*- and *trans*-regulatory differences acting in opposite directions resulting in no net expression difference between species) (Fig. 2*C*).

Of 4,671 genes with regulatory changes between human-only and chimpanzee-only iPSC lines, 44.4% (2,073 genes) were regulated primarily in *cis*, 31.4% (1,465 genes) were regulated primarily in *trans*, and the remaining 1,133 genes were regulated both in *cis* and in *trans* (Fig. 2*C*). This final category was further broken down into a *cis+trans* category (*cis*- and *trans*-regulatory changes acting in the same direction) and a *cis–trans* category (*cis*- and *trans*-regulatory changes acting in opposite directions). This yielded 20.6% (961 genes) and 3.7% (172 genes) regulated in *cis+trans* and *cis–trans*, respectively. Other genes that did not satisfy the conditions for any category (3,515 genes) were classified as ambiguous.

Genes with primarily *cis*-regulatory changes had a larger median effect size than genes with primarily *trans*-regulatory changes (median $\left|log_{2}(FC)\right|$ of 1.09 vs. 0.64, $P<10^{-56}$Z(HTML translation failed) by two-tailed Mann–Whitney *U* test; Fig. 2*D*). Genes classified as *cis+trans* had the highest effect size of any regulatory type category (median $\left|log_{2}(FC)\right|$ of 1.21).

Gene ontology enrichments for genes classified as *trans* included processes related to the skeletal, cartilage, and muscular systems (Dataset S6). Although we did not assess gene expression differences in skeletal, cartilage, or muscle cells, previous studies that assessed regulatory differences between human and chimpanzee embryonic stem cells similarly found gene ontology enrichments associated with differentiated tissues, including the vocal tract (18). The enrichments seen in the current experiments suggest that some of the dramatic skeletal and muscular differences between humans and chimpanzees may be driven by *trans*-acting regulatory changes. Additionally, genes classified as conserved had gene ontology enrichments related to voltage-gated ion channels (Dataset S6), which are important for maintaining critical features of iPSCs, including proliferation capacity and differentiation potential (19). Finally, genes classified as compensatory had enrichments related to ligase activity, neurexin protein binding, and phosphatidylserine binding, while all other regulatory type classifications had no significant gene ontology enrichments.

We also used the Gene ORGANizer database (20), which links genes to the body parts they affect based on phenotypes associated with Mendelian disorders, to test whether genes that were differentially expressed between humans and chimpanzees tend to influence more or fewer biological systems than conservedgenes. We found that genes with primarily *cis*-regulatory changes and at least a twofold change in expression influenced a median of 18 body parts compared to a median of 30 body parts influenced by conserved genes (adjusted $P=2.8\times 10^{-4}$ by two-tailed Mann–Whitney *U* test; Fig. 2*E*). Interestingly, the greater the expression differences between human and chimpanzee as indicated by higher $\left|log_{2}(FC)\right|$, the fewer body parts a gene with primarily *cis*-regulatory changes tended to influence (*SI Appendix*, Fig. S3). Similar trends were observed for genes classified as *cis*+*trans* but not other regulatory categories.

Removing reads mapping to genes on chromosomes that were karyotypically abnormal in any of our iPSC lines did not significantly change our regulatory type classification or effect size results (*SI Appendix*, Fig. S4). Together, our results indicate that both *cis*- and *trans*-acting regulatory changes are important contributors to the widespread gene expression differences between humans and chimpanzees in iPSCs, with *cis*-regulatory changes tending to be larger and to act on genes affecting fewer biological systems (Fig. 2 *C–E*).

### Prospects for Genetic Mapping.

Further localization of both *cis*- and *trans*-regulatory differences would be greatly aided if it were possible to generate mapping panels that carry different dosages of human and chimpanzee alleles at known locations throughout the genome. Previous studies in yeast, *Drosophila*, and cultured mammalian cell lines have used mitotic recombination to generate useful mapping panels from somatic cells (11, 12, 21, 22). To boost the rate of mitotic recombination, common strategies have been to treat cells with small molecules that promote DNA damage (23, 24), or to induce targeted recombination at specific loci using CRISPR-Cas9 (12, 21).

**Fig. 3.**
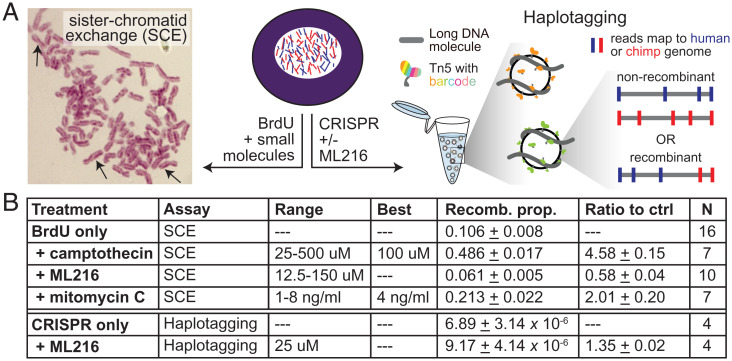
Effect of small molecules on chromosome recombination frequencies in allotetraploid cells. The small molecules camptothecin, ML216, and mitomycin C were assessed for their effect on intraspecific and interspecific recombination. (*A*) Allotetraploid cells (*Center*) were treated with BrdU and small molecules to measure intraspecific SCE events by microscopy (*Left*), or treated with Cas9 and gRNAs with or without ML216 followed by haplotagging to identify interspecific recombinant molecules by sequencing (*Right*) (*Materials and Methods*). (*B*) The proportion of chromosomes that had SCE events after treatment with the indicated concentrations of each small molecule is listed (recomb. prop.). N denotes number of replicate experiments where the recomb. prop. was quantified for all concentrations or the best concentration (when indicated). Note that BrdU was added to all cells to visualize SCE, and all BrdU+drug treatments were compared to the BrdU-only condition (ratio to ctrl). For haplotagging, the effect of CRISPR guides targeting specific loci was assessed with or without ML216. Compared to CRISPR alone, ML216 may elevate the proportion of interspecific recombinant molecules genome wide (ratio to ctrl). Values are mean ± SEM.

To assess whether small molecules could stimulate mitotic cross-overs in iPSCs, we performed sister chromatid exchange (SCE) assays by incubating cells with BrdU for two cell cycles (25). Chromosomes where both strands incorporate BrdU stain lighter than chromosomes where only one strand has incorporated BrdU, making it possible to visualize SCE events in mitotic chromosome spreads (Fig. 3*A*). We tested camptothecin, a topoisomerase inhibitor previously found to induce SCE events in iPSCs (23). Consistent with prior findings, treatment of 100 nM camptothecin for 1 h induced a 4.5-fold increase in SCE events in both autotetraploid and allotetraploid iPSCs ($P<10^{-8}$ by one-tailed Student’s *t* test; Fig. 3*B*).

We also tested ML216, a BLM inhibitor, which has been found to induce SCE events in cultured human cells (9, 10, 24). However, we found that treatment with ML216 over a range of concentrations from 12.5 µM to 150 µM did not increase the rate of SCE events in iPSCs. We additionally tested mitomycin C, which cross-links DNA and is known to induce SCE events in yeast and fungi (26). Treatment of 4 ng/mL mitomycin C for 24 h in tetraploid iPSCs increased the rate of SCE events twofold ($P<10^{-5}$ by one-tailed Student’s *t* test; Fig. 3*B*). Although SCE assays can only reliably assess intraspecific cross-over events, these results suggest that the application of camptothecin or mitomycin C to allotetraploid iPSCs has the potential to similarly increase the rate of interspecific mitotic recombination.

An alternate approach is to induce targeted cross-overs using CRISPR-Cas9. This strategy has previously been used to induce recombination in yeast and *Drosophila* (12, 21). To determine the rate of interspecific recombination events at target loci, we used a recently developed technique called haplotagging to directly detect recombinant junctions by barcoding DNA molecules prior to sequencing (27, 28). Following sequencing, reads were aligned to a composite human–chimpanzee genome and comparatively assigned to their species of origin. Reads derived from the same DNA molecule were tagged with the same barcode, enabling molecule reconstruction (*Materials and Methods*). Barcoded molecules that mapped to orthologous intervals in human and chimpanzee and showed switched runs of variants from one species to the other (human to chimpanzee, or vice versa) were scored as likely interspecific recombination events within the corresponding genomic interval.

In the allotetraploid line H1C1a, we targeted genomic loci on chr20q13.33, chr21q22.3, and chrXq28 with CRISPR gRNAs and then performed haplotagging to over 200× molecular coverage (*SI Appendix*, Figs. S5 and S6). Based on a recent study suggesting that ML216 acts synergistically with CRISPR-Cas9 to induce loss of heterozygosity at targeted loci in human iPSCs (22), we also assessed whether the addition of 25 µM of the BLM inhibitor ML216 starting 12 h before gRNA targeting and ending 48 h posttargeting would affect the rate of interspecific recombination. We did not observe an enrichment in interspecific recombination events at any of the target loci with or without ML216 treatment (*SI Appendix*, Fig. S6). However, genome-wide interspecific recombination events trended 1.35-fold higher when comparing ML216-treated samples against samples that were only treated with CRISPR-Cas9 (*P* = 0.052 by one-tailed paired Student’s *t* test; Fig. 3*B*). After ML216 treatment for 60 h (approximately three cell divisions), we detected a total of 878 interspecific recombination events in ∼83 million analyzed molecules. This translates to an endogenous rate of ∼0.8 recombination events per cell per generation and an increased rate of approximately one recombination event per cell per generation after ML216 treatment. These apparent rates in allotetraploid cells are substantially higher than previously reported mitotic recombination rates in diploid human and mouse embryonic stem cells [0.01 to 0.04 recombination events per cell per generation after ML216 treatment; *SI Appendix*, *Supplemental Materials and Methods* (10, 11, 29)]. Further investigation will be required to assess whether ML216 significantly increases the rate of interspecific recombination and whether other small molecules such as camptothecin can also increase the rate of recombination events between human and chimpanzee chromosomes in allotetraploid iPSCs.

### Targeted *cis*- and *trans*-Mapping on the X Chromosome.

**Fig. 4.**
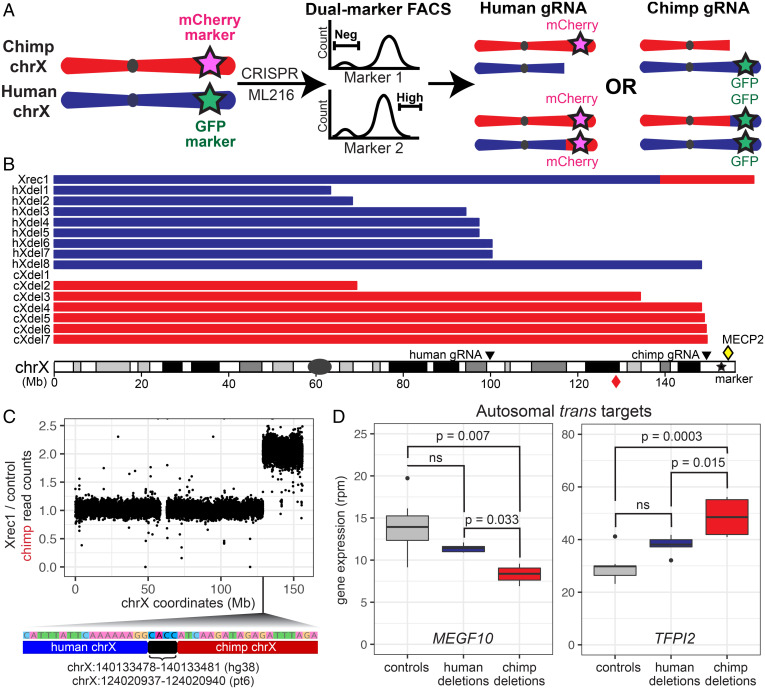
X chromosome targeting generates recombinant and deletion lines for genetic mapping. Cell lines with recombination and deletion events on the X chromosome were isolated by FACS and used for further mapping of regulatory sequences. (*A*) Allotetraploid cells marked with GFP on the distal human chrX and mCherry on the distal chimpanzee chrX were treated with ML216, Cas9, and species-specific chrX gRNAs, followed by FACS for expected signatures of deletion or recombination (loss of fluorescent marker on targeted chrX, or retention or gain of fluorescent marker from homologous, untargeted chrX). (*B*) Cell lines recovered from sorting contained either a recombinant chrX or species-specific distal chrX deletions. Breakpoint locations for the recombinant (Xrec1), human deletions (hXdel#), and chimpanzee deletions (cXdel#) are shown relative to human chrX. Positions of species-specific gRNAs are shown. Red diamond symbol indicates the distal portion of chimpanzee chrX that is recombined onto the first 140.1 Mb of human chrX in Xrec1. Yellow diamond symbol indicates the position of *MECP2* on human chrX. (*C*) Whole-genome DNA sequencing of Xrec1 and a control sample (X1-S; *Materials and Methods*) showed an increase in chimpanzee read depth ratio along the X chromosome and identified human–chimpanzee spanning sequence reads at the point of transition, locating the precise point of cross-over for a human–chimpanzee recombinant X chromosome (position along chrX shown in hg38 coordinates; bracket indicates 4 bp of microhomology found in both human and chimpanzee chrX at the indicated coordinates). (*D*) Expression of autosomal genes *MEGF10* and *TFPI2* was significantly different in four lines that have lost distal chimpanzee chrX sequences (cXdel4–cXdel7) when compared to nine control lines without deletions or to five lines that have lost distal human chrX sequences (hXdel3–hXdel7), as expected if a *trans*-regulatory factor that differs between humans and chimpanzees maps to distal chrX (*SI Appendix*, *Supplemental Materials and Methods*).

To further enrich for cells that may contain interspecific mitotic recombination events at specific loci, we fluorescently marked allotetraploid cells at distal chromosome ends. This allowed us to use FACS to isolate cells with expected signatures of recombination (Fig. 4*A*). The gene density and enrichment of disease-related genes on distal chrX, particularly chrXq28, made the distal region of chrX a particularly attractive target for further study (30). Through two rounds of CRISPR-Cas9–mediated homologous recombination (HR), we generated five allotetraploid lines derived from H1C1a and one from H2C2b, each carrying *GFP* on the human chrX and *mCherry* on the chimpanzee chrX (*SI Appendix*, Fig. S7). Some allotetraploid cells that underwent two rounds of CRISPR-Cas9 HR insertion maintained largely normal karyotypes, while others showed more extensive aneuploidies (Dataset S1).

We then targeted the double fluorescently marked lines with species-specific gRNAs to induce interspecific recombination events on the X chromosome. Because the allotetraploids were derived from fusions of male cells, only one X chromosome was present from each species. In the double fluorescently marked lines, cells with no recombination events on chrX should carry both human and chimpanzee fluorescent markers, while recombinant cells should carry two copies of a single marker from either human or chimpanzee chrX. We treated the double fluorescently marked allotetraploid line H1C1a-X1 with either a chimpanzee-specific gRNA targeting chrXq28 or a human-specific gRNA targeting chrXq22.1, in combination with 25 µM ML216 (*SI Appendix*, Fig. S8 and *Materials and Methods*).

Cells targeted with the chimpanzee-specific gRNA were sorted for the absence of mCherry, which marks the chimpanzee chrX, and increased intensity of GFP, to select for likely recombination events that result in two human alleles on the distal end of chrXq. Because we observed higher fluorescence intensity of the human chrX marker GFP in untreated cells during the G2/M cell cycle phase, we also used Hoechst DNA staining to sort specifically from G1 cells in the experiments with the chimpanzee-specific gRNA (*SI Appendix*, Fig. S9 and *Materials and Methods*). Similarly, cells targeted with the human-specific gRNA were sorted for the absence of GFP, which marks the human chrX, and increased intensity of mCherry. For the human-specific gRNA sorts, we also incorporated an additional marker by staining for a linked cell-surface protein, TSPAN6. Located on chrXq, *TSPAN6* has 1.4-fold higher *cis*-regulated expression from the chimpanzee allele compared to the human allele (adjusted $P=1.4\times 10^{-4}$ by Welch’s *t* test); protein staining of TSPAN6 showed a similar difference (*SI Appendix*, Fig. S9 and *Materials and Methods*). Cells targeted with human-specific gRNA were thus sorted for absence of human marker GFP and increased intensity of both chimpanzee marker mCherry and of TSPAN6.

A total of 951 allotetraploid candidate colonies were grown from single cells after FACS. Additional genotyping confirmed that 172 colonies carried distal chrXq from a single species (Dataset S7). As expected, in 172/172 (100%) of these cases, the missing chrXq corresponded to the species targeted by the gRNA (79/79 for the human gRNA, and 93/93 for the chimpanzee gRNA). To distinguish between deletion and recombination events, we determined the relative dosage of chrXq in these colonies by performing qPCR assays on genomic DNA at chr6p, chrXp, and chrXq (Dataset S2 and *SI Appendix*, *Supplemental Materials and Methods*). We found that 171/172 (99.4%) colonies had lost the distal end of chrX of one species without altering the chrX dosage of the other species, as expected if targeting of the X chromosome had produced a species-specific deletion in these colonies.

We also identified one colony (0.6%) that had not only lost the distal end of the chimpanzee chrX but also doubled the dosage of the distal end of human chrX, consistent with a possible recombination event. Whole-genome sequencing of this putative recombinant line confirmed that it was an interspecific recombinant, with the first 140.1 Mb of human chrX fused to the distal 27.6 Mb of chimpanzee chrX (Xrec1 in Fig. 4 *B* and *C* and *SI Appendix*, Fig. S10). Sequence reads that span the precise junction between the human and chimpanzee sequences show that recombination did not occur in a region of large-scale homology between the two X chromosomes. Instead, a 4 bp microhomology occurs directly at the junction site, suggesting that the recombination event was likely produced by microhomology-mediated end joining and not by HR (31). No other human–chimpanzee recombinant chromosomes were found in the sequenced Xrec1 line when compared to an untreated control line, suggesting that recombination events elsewhere in the genome are rare in cells that survive chrX targeting, FACS, and plating and growth of colonies from single cells.

We next leveraged the species-specific targeted lines as a panel of deletion lines for fine-mapping studies. We performed bulk RNAseq on seven lines with partial chimpanzee chrX deletions, eight lines with partial human chrX deletions, and nine control lines without chrX deletions (Dataset S1). We identified the approximate breakpoint of each deletion by examining the ratio of reads that uniquely map to either the human or the chimpanzee genome along chrX (Fig. 4*B*, *SI Appendix*, Fig. S11, and *Materials and Methods*). In every case, mapped breakpoints were consistent with our results from genomic PCR and qPCR assays (Dataset S7). Three of the seven chimpanzee chrX deletions mapped within 1 Mb of the chimpanzee-specific gRNA target site, and two of the eight human chrX deletions mapped within 1 Mb of the human-specific gRNA target site (Fig. 4*B*). The remaining lines had a range of breakpoints that were up to 80 Mb away from the species-specific guide targeting sites, and one cell line appeared to have lost the targeted X chromosome completely (Fig. 4*B*).

To further characterize the breakpoints in X chromosome deletion lines, we performed whole-genome DNA sequencing of cXdel5 and cXdel6, which were plated and expanded from single cells after FACS selection. DNA sequencing indicated that cXdel5 cells had lost chimpanzee sequences distal to 147 Mb, retained chimpanzee sequences proximal to 140 Mb, and likely contained a subclonal mixture of deletion breakpoints in between. The cXdel6 cells had lost chimpanzee sequences distal to 148 Mb, retained sequences proximal to 147 Mb, and likely contained a subclonal mixture of insertions in between (*SI Appendix*, Fig. S12*A*). CRISPR targeting can thus induce terminal chromosome deletions, with staggered endpoints forming in the region around the breakpoints.

The X chromosome breakpoints in cXdel5 and cXdel6 cells were located near the genes *FMR1* and *AFF2*. To test whether species-specific chromosome deletions cause species-specific changes in gene expression near the breakpoints, we examined the level of expression of the human and chimpanzee alleles of *FMR1* and *AFF2* in cXdel4, cXdel5, cXdel6, cXdel7, and control cells. The human alleles of *FMR1* and *AFF2* showed normal expression in the chimpanzee chrX deletion lines compared to control cells. In contrast, the chimpanzee alleles of *FMR1* and/or *AFF2* were not expressed when the genes were located distal to the deletion breakpoint in cXdel4, showed reduced or absent expression when the genes were located in the region of staggered deletions in cXdel5, and showed normal expression when the genes were located proximal to the terminal deletions in cXdel6 and cXdel7 (*SI Appendix*, Fig. S12*B*). Chimpanzee-specific chrX deletions thus can disrupt gene expression in *cis* without resulting in compensatory up-regulation of the corresponding human allele on the remaining X chromosome.

If the X chromosome encodes *trans* regulators of autosomal gene targets, partial deletions of either the human or chimpanzee X chromosome could result in significant gene expression changes for genes located on autosomes. Indeed, 42 autosomal genes showed significant changes in expression in the four deletion lines that removed regions on the chimpanzee X chromosome distal to breakpoints around 148 Mb when compared to control lines without chrX deletions, and even more autosomal genes (147) showed significant changes in expression in the five cell lines that removed regions on the human X chromosome distal to breakpoints around 95 Mb (Dataset S8). Interestingly, seven of the genes altered by loss of distal chimpanzee chrX regions were not significantly different in the cell lines that had lost even larger regions of the human X chromosome. These genes also showed the expected signatures of a species-specific *trans* effect when comparing gene expression levels among the different deletion lines (*SI Appendix*, *Supplemental Materials and Methods*). The autosomal genes included *MEGF10* and *TFPI2*, which were both classified as having a significant *trans* component in our studies of intact diploid, autotetraploid, and allotetraploid cells (Fig. 4*D* and Dataset S5). The magnitude of differential expression seen after species-specific removal of the distal X chromosome ranged from 60 to 80% of the overall expected *trans* component. Thus, *trans* regulators encoded on the X chromosome may contribute to a fraction of the species-specific *trans*-expression differences observed in these autosomal genes. Extensions of this approach could be used to further localize the responsible *trans* factors on the X chromosome, as well as *trans* factors on other chromosomes.

## Discussion

Understanding the molecular basis of human evolution is a grand and ambitious challenge in biological research. At the molecular level, researchers have cataloged the DNA sequence changes between humans and nonhuman primates (5) and identified many RNA expression differences between humans and chimpanzees across multiple tissues and developmental stages (13, 32, 33). However, it has been difficult to map the exact sequence changes that cause particular gene expression differences or other species-specific traits. Here, we have used intraspecific and interspecific iPSC fusions to determine whether human–chimpanzee gene expression changes are controlled in *cis* or *trans*, and have developed genetic methods for further mapping both *cis* and *trans* effects to particular locations in the genome.

Regulatory changes appear to be a key driver of evolution in humans and other systems (34), and we and others have worked to determine the relative contribution of *cis*- and *trans*-acting regulatory changes to gene expression differences between species. As in previous studies with human and chimpanzee iPSCs (13, 14), we found thousands of genes with species-specific expression differences. By comparing DE in single-species and cross-species fusions, we found that (1) both *cis*- and *trans*-regulatory changes are key contributors to human–chimpanzee differences, and (2) genes with *cis*-regulatory changes had, on average, more divergent expression than genes with *trans*-regulatory changes. Both of these findings are consistent with previous genome-wide studies of human–chimpanzee tetraploid cortical spheroids, human–chimpanzee tetraploid cranial neural crest cells, and interspecific hybrids of mice, maize, *Arabidopsis*, and yeast (14, 15, 17, 35).

We also found that genes with *cis*-regulatory changes tended to influence fewer body parts than genes conserved between human and chimpanzee iPSCs. Furthermore, the number of body parts affected by a gene declines as the expression difference between humans and chimpanzees increases. *cis*-regulatory changes are often thought to be favored in evolution because of their ability to avoid negative pleiotropy and restrict changes to particular tissues (34, 36). Our data suggest that genes that influence fewer biological processes are also more likely to evolve large expression differences as species diverge during evolution.

To facilitate further genetic mapping of human–chimpanzee differences, we examined multiple strategies to induce recombination events in allotetraploid iPSCs, including both genome-wide and targeted approaches. The BLM inhibitor ML216 has been successfully used to induce interchromosomal recombination in other systems (11, 22). In our experiments, ML216 treatments did not cause a measurable increase in intrachromosomal exchange events scored by SCE assays, but may have stimulated a modest ∼35% increase in the number of human–chimpanzee recombinant molecules identified by haplotagging (Fig. 3). These differences could be nonsignificant, or might result from molecular differences between intraspecific and interspecific recombination events; confounding effects of BrdU, which promotes DNA damage and differentiation (*SI Appendix*, Fig. S13), in the SCE assay (37); or synergistic effects with CRISPR-Cas9 targeting, as previously reported for other mitotic recombination assays in human iPSCs (22). Further varying BLM activity by either pharmacological or genetic strategies (9, 10) or by treating with ML216 for multiple passages could be tested for larger effects on the overall rate of recombination. Camptothecin and mitomycin C treatments are also promising candidates for further study, given their strong promotion of SCE events in allotetraploid iPSCs (Fig. 3*B*).

We also tested the ability of Cas9 and gRNAs to stimulate interspecific chromosome exchange events at particular locations in the genome. In contrast to prior work in yeast, *Drosophila*, and human iPSCs (12, 21, 22), we did not observe an enrichment in interspecific recombination events at the site of targeting with or without ML216 by analyzing bulk populations with haplotagging. We also did not recover recombination events at the site of CRISPR targeting in the fluorescently marked lines that we sorted to enrich for signatures of rare recombination events on the X chromosome. We did recover many lines that carried species-specific X chromosome deletions with breakpoints near the site of CRISPR targeting. We further recovered a single line carrying a confirmed human–chimpanzee recombinant X chromosome. However, the cross-over junction in the recombinant line was located tens of megabases away from the CRISPR targeting site and may be the result of a spontaneous or ML216-induced, rather than a CRISPR-induced, breakpoint on the X chromosome.

Our overall rates of recovering targeted X chromosome changes in allotetraploid lines were low (from ∼78 million input cells, 951 colonies survived FACS selection and plating, of which a single colony contained a recombinant chromosome and 171 colonies contained deletion chromosomes). We note that estimated rates of interspecific recombination appeared orders of magnitude higher when bulk cells were analyzed by haplotagging shortly after ML216 treatment (approximately one genome-wide interspecific recombination event per cell per generation). Interspecific recombination rates in allotetraploid cells may be overestimated by haplotagging, due to barcode sharing between DNA molecules or errors in assigning reads to the correct species when comparing allotetraploid cells with reference genomes. Alternatively, high rates of interspecific recombination may be incompatible with long-term growth and survival in allotetraploid cells such that only cells with low numbers of recombinant chromosomes survive FACS selection, plating, and growth at clonal density after treatments. Despite the low overall rate of recovering useful cells in the targeting experiments, our experiments show that informative panels can be successfully generated by treating large numbers of cells and selecting for changes on particular chromosomes.

A variety of strategies may make it possible to increase the rate of homology-directed interspecific recombination. Following induction of double-strand breaks by small molecules or Cas9, homology-directed repair (HDR) pathways compete with several other pathways, including nonhomologous end joining (NHEJ) (38). Studies in other systems have shown that the HDR pathway can be stimulated by expressing a plasmid with *RAD18*, a gene involved in the DNA damage response, or by treating cells with the small-molecule RS-1 which increases the activity of the HDR-promoting protein RAD51 (39, 40). Conversely, the competing NHEJ pathway can be suppressed using the small-molecule Scr7 to inhibit DNA Ligase IV, a key component of NHEJ (39). Studies in yeast show that tethering Cas9 to Spo11, a DSB-inducing protein with a key role in initiating meiotic recombination, can stimulate cross-overs in naturally recombination-cold regions (41). These and other approaches can now be tested for their ability to stimulate targeted recombination between human and chimpanzee chromosomes in allotetraploid cells.

Like genetic mapping using recombinants, deletion mapping has also been used to map phenotypes to specific genomic regions in many organisms (42, 43). Our targeting and sorting strategies have already successfully produced a panel of deletion lines useful for further mapping of *cis* effects and *trans*-acting factors on the X chromosome. The fraction of the genome removed by the induced chrX deletions is similar to the fraction of the genome removed by typical deficiency mapping chromosomes in *Drosophila* [0.2% of the genome deleted, on average (43)]. The staggered deletions that form after chromosome targeting, both in different colonies and within the same colony (e.g., cXdel5) after FACS selection, could be harnessed for further fine mapping of *cis*-regulatory effects in a chromosomal region of interest.

Panels of chromosome deletion lines can also be used to map species-specific *trans* regulators. *trans* effects appear to contribute to more than 50% of the gene expression differences identified between humans and chimpanzees in iPSCs (Fig. 2*C*), and are similarly pervasive in other systems (14, 15, 17, 35, 44). Our targeted X chromosome deletion lines suggest that human–chimpanzee differences in the autosomal genes *MEGF10* and *TFPI2* are controlled, in part, by species-specific *trans* effects that map to the most distal ∼8 Mb of the X chromosome. One of the genes located in this distal X chromosome region is *MECP2*, which encodes a methyl DNA-binding protein that can activate or repress expression of target genes (45). Loss-of-function mutations in *MECP2* lead to Rett syndrome, a severe neurodevelopmental disorder. Intriguingly, prior research has identified both *MEGF10* and *TFPI2* as genes regulated by MeCP2 in human cells (46, 47). Further, both MEGF10 and MeCP2 have been linked to the pruning of neural synapses by astrocytes (48, 49), a cell type that has undergone changes in number, spatial organization, and function during human evolution (50, 51). Given that gene regulation in iPSCs cells has been shown to be similar to that in somatic tissues in some contexts (52), it is tempting to speculate that this potential *trans* regulation might contribute to human–chimpanzee astrocyte differences or changes in neural processes and circuits pruned by astrocytes. Future experiments to selectively knock out either the human or chimpanzee *MECP2* allele could test whether MeCP2 indeed regulates the species-specific expression of *MEGF10* and *TFPI2* in iPSCs, as well as potentially identify other species-specific *trans* targets for this key transcriptional regulator.

We have focused most of our current studies on gene expression differences that are detectable in undifferentiated iPSCs. It is possible that tetraploidization will disrupt gene expression or limit the differentiation potential of autotetraploid and allotetraploid iPSC lines. However, previous studies have shown that tetraploid mouse embryos can form most major organs, and rare humans with tetraploid karyotypes have been reported to survive for up to 2 y after birth (53, 54). In addition, our global RNA profiling experiments showed no large-scale gene expression disruptions between diploid and autotetraploid lines. We also find that diploid lines are more similar to their cognate autotetraploid lines than to other diploid lines of the same species. Thus, diploid and tetraploid iPSCs appear remarkably similar at the gene expression level. Future studies will be needed to determine whether this similarity is maintained under a variety of differentiation conditions. Our initial experiments show that diploid, autotetraploid, and allotetraploid cells can all express characteristic gene markers of ectoderm, mesoderm, or endoderm under appropriate differentiation conditions (Fig. 1*C* and Dataset S3), and other tetraploid fusion lines have recently been differentiated into cortical spheroids or neural crest cells in vitro (14, 15). We caution that some of the lines in our own experiments showed incomplete endoderm differentiation, and previously reported allotetraploid lines showed substantial expression of mesenchymal markers when incubated under conditions that stimulate cortical spheroid formation in diploids (14). In vitro differentiation protocols may thus need to be altered or optimized for tetraploid iPSCs to find conditions suitable for formation of particular cell types of interest.

We have found that tetraploid iPSCs can be grown, repeatedly passaged, tagged with fluorescent markers, and subcloned while maintaining grossly normal karyotypes. Whole-genome sequencing of Xrec1 after CRISPR-Cas9 targeting of chrX shows that induced changes also occur specifically on the targeted chromosome of interest. However, we have also found karyotypic abnormalities in some cell lines when multiple subclones are expanded from a particular cell fusion or treatment (Dataset S1). DNA sequencing further shows that heterogeneity may exist within a colony grown from single cells, such as the staggered breakpoints occurring on the X chromosome in cXdel5 and cXdel6 (*SI Appendix*, Fig. S12). At times, it may be possible to put such heterogeneity to experimental advantage. For example, the staggered deletions occurring within cXdel5 cells may make it possible to establish a larger panel of subclones that could be used for additional fine mapping of *cis* and *trans* factors on the X chromosome, all derived from a single initial round of targeting. However, further study of karyotypic and chromosomal stability in tetraploid iPSC lines is clearly warranted, and we recommend that interested researchers continue to monitor key cell lines and derivatives using periodic karyotyping and whole-genome sequencing approaches.

Beyond mapping the *cis* and *trans* regulators of species-specific gene expression differences, we envision that allotetraploid iPSC lines will also be useful for mapping cellular and tissue differences between humans and chimpanzees. For example, many metabolic differences have evolved alongside major changes in diet between humans and chimpanzees (55). These changes are likely accompanied by cellular changes in enzyme levels and metabolite production that could be scored under appropriate in vitro conditions. In addition, neural progenitors in humans have been shown to have a longer prometaphase and longer metaphase compared to those in chimpanzees (56). These and other cellular traits can be assessed in culture and are compelling candidates for allotetraploid genetic mapping approaches. Recent advances in organoid technology also make it possible to study organ-level phenotypes that differ between humans and chimpanzees, including differences in organ size, connectivity, and cell type composition (33). Just as meiotic mapping panels have propelled our understanding of evolution in other organisms, further development of mapping methods in human–chimpanzee allotetraploids should provide powerful new genetic approaches for our quest to understand what makes us human.

## Materials and Methods

### Generation and Maintenance of Tetraploid iPSC Lines.

Human and chimpanzee diploid iPSC lines were labeled with diffusible fluorescent dyes and fused on an Eppendorf Multiporator at 4-V AC for 80 s, 16-V DC for 20 µs, and 6-V post-AC for 95 s (*SI Appendix*, *Supplemental Materials and Methods*). Tetraploid lines were confirmed by propidium iodide staining and karyotyping (Dataset S1). Diploid and tetraploid iPSC lines were routinely propagated feeder-free (*SI Appendix*, *Supplemental Materials and Methods*).

### Trilineage Differentiation.

Diploid and tetraploid iPSC lines were differentiated with the STEMdiff Trilineage Differentiation Kit according to the manufacturer’s instructions (STEMCELL Technologies, catalog #05230). Differentiation was assessed using qRT-PCR for pluripotency, ectoderm, mesoderm, and endoderm gene markers (Dataset S2 and *SI Appendix*, *Supplemental Materials and Methods*).

### RNAseq Analysis.

Sequencing reads were aligned to a composite human–chimpanzee genome (hg38 and pt6), and the number of uniquely mapped reads that overlap each gene was determined using a curated exon annotation (*SI Appendix*, *Supplemental Materials and Methods*). DE analysis between diploids and autotetraploid iPSCs was performed with DESeq2 (57), and genes with an adjusted *P* < 0.05 and at least a twofold change in expression were called as significant (*SI Appendix*, *Supplemental Materials and Methods*).

DE between single-species iPSCs, ASE in allotetraploids, and regulatory type classifications were carried out as a combination of previously described methods (35, 44). Genes were classified as *cis*, *trans*, *cis+trans*, *cis–trans*, compensatory, conserved, or ambiguous based on different combinations of significant DE, significant ASE, significant $log_{2}(FC)$ difference between DE and ASE (“*trans* effects”), and direction of *cis* contribution and *trans* contribution to the DE $log_{2}(FC)$ (*SI Appendix*, *Supplemental Materials and Methods*).

### SCE Assay.

Camptothecin (Sigma Aldrich, catalog #C9911-100MG), ML216 (Cayman Chemical, catalog #15186), and mitomycin C (Sigma Aldrich, catalog #M4287-2MG) were applied to iPSCs with 10 µM BrdU (*SI Appendix*, *Supplemental Materials and Methods*). The SCE assay was then performed as previously described (25).

### Haplotagging.

Haplotagging was performed as previously described (28). Reads were aligned to a composite human–chimpanzee genome (hg38 and pt6) and assigned to their molecule of origin by barcode. Variants between hg38 and pt6 were identified for each read and filtered by multiple criteria (*SI Appendix*, *Supplemental Materials and Methods*). Molecules were scored as recombinant if they contained one interspecific event and approximately five supporting variants per species.

### FACS of Fluorescently Marked Allotetraploid Lines.

Using two rounds of HR, we inserted an EF1a-EGFP-IRES-PuroR cassette at human chrXq28 and an EF1a-mCherry-IRES-NeoR cassette at chimpanzee chrXq28 in allotetraploid iPSCs (*SI Appendix*, Fig. S7 and *Supplemental Materials and Methods*). Double-marked iPSCs were treated with 25 µM ML216 starting 12 h before nucleofection of CRISPR-Cas9 and gRNA and continuing until 48 h postnucleofection. We then employed multiple sorting strategies to enrich for chrX recombination or deletion events (*SI Appendix*, Fig. S9 and *Supplemental Materials and Methods*).

### DNA Sequencing Analysis of chrX Recombinant and Deletion Lines.

DNA sequencing reads from the recombinant allotetraploid cell line, two chimpanzee chrX deletion lines, and a control allotetraploid line were aligned to a composite human–chimpanzee (hg38–pt6) reference genome (*SI Appendix*, *Supplemental Materials and Methods*). The read counts in the recombinant or deletion lines were normalized to read counts in the control line (*SI Appendix*, Figs. S10 and S12).

## Supplementary Material

Supplementary File

Supplementary File

Supplementary File

Supplementary File

Supplementary File

Supplementary File

Supplementary File

Supplementary File

Supplementary File

## Data Availability

Data supporting the findings of this study are included in the main text and *SI Appendix* or deposited in publicly available databases. RNAseq data generated in this study are available at Gene Expression Omnibus (GSE184768) (58), and the DNA sequence containing the recombination site for H1C1a-X1-Xrec1 is available at GenBank (OK283040) (59). Additional materials will be made available upon request.

## Change History

January 21, 2022: The byline has been updated.
