# Supplementary material for: Genetic studies of human–chimpanzee divergence using stem cell fusions
Source: Proc Natl Acad Sci U S A. 2021 Dec 17;118(51):e2117557118. doi: 10.1073/pnas.2117557118 (PMC8713981; doi:10.1073/pnas.2117557118)
Supplement: Supplementary File [file pnas.2117557118.sapp.pdf]

PNAS
www.pnas.org

Supplementary Information for

Genetic studies of human-chimpanzee divergence using stem cell fusions

J.H.T. Song, R.L. Grant, V.C. Behrens, M. Kucka, G.A. Roberts Kingman, V. Soltys, Y.F. Chan, D.M. Kingsley

David M. Kingsley
E-mail: kingsley@stanford.edu

This PDF file includes:

Supplementary text
Figs. S1 to S13
Legends for Dataset S1 to S8
SI References

Other supplementary materials for this manuscript include the following:

Datasets S1 to S8

Supporting Information Text

Supplemental Materials and Methods

Cell culture maintenance. The induced pluripotent stem cell lines H23555 (H1), H20961 (H2), C3649 (C1), and C8861 (C2) were provided by the Gilad laboratory (1). Cultures were tested for and maintained mycoplasma free. Diploid and tetraploid lines were routinely propagated feeder-free in mTeSR1 or mTeSR Plus media (STEMCELL Technologies, cat #85850 and cat #100-0276) on cell culture plastics coated with Geltrex basement membrane matrix (Gibco, cat #A1413302). When confluent, cells were passaged using Accutase (Millipore, cat #SCR005) with 1μM thiazovivin (Tocris, cat #3845) or using 0.5mM EDTA as previously described (2, 3). Cells were imaged on the EVOS FL microscope (Thermo Fisher) at 4X magnification, unless otherwise noted.

Generation of tetraploid iPSC lines. One diploid iPSC line was labeled with CellTracker Green CMFDA Dye (1:667) (Thermo Fisher, cat #C7025) and the other diploid iPSC line was labeled with CellTracker Blue CMAC dye (1:500) (Thermo Fisher, cat #C2110) or CellTracker Red CMTPX Dye (1:1000) (Thermo Fisher, cat #C34552) per the manufacturer's instructions. Cells were washed multiple times to remove excess dye and allowed to recover after labeling in mTeSR1 + 1μM thiazovivin for at least 1 hour prior to fusion. $7x10^5$ cells from each line were combined, washed twice in 1ml fusion buffer, resuspended in 350μl fusion buffer, and fused in the helix fusion chamber of an Eppendorf Multiporator at 4V AC for 80s, 16V DC for 20μs, 6V post-AC for 95s. After 10 minutes at room temperature, 1ml of mTeSR1 + 1μM thiazovivin was added to the helix fusion chamber. Fusion buffer was hypoosmolar electrofusion buffer (Eppendorf, cat #940002150) diluted in water (normally, 60% hypoosmolar electrofusion buffer and 40% water).

Tetraploid clones were then selected in one of two ways. In the first method, 250-350μl of the resulting suspension after fusion was immediately plated in a 10-cm plate. Double-labeled cells were screened the following day under a fluorescent microscope and marked on the plate. The diffusible dyes were only visible for 2 days after fusion. Surrounding diploid cells were removed on each subsequent day by manual scraping. When the originally identified double-labeled cells grew into colonies, they were picked into a 96-well plate and screened as described below.

In the second method, 7-15 fusions were performed on the same day using the helix fusion chamber as described above and collected in a large volume of mTeSR1 + 1μM thiazovivin for fluorescence-activated cell sorting (FACS). For increased viability in the second method, we used only CellTracker Green CMFDA and CellTracker Blue CMAC dyes. Cells were gently pipetted every hour to avoid CellTracker dye diffusion and undesired labeling of nearby non-fused cells. Prior to FACS, cells were resuspended in 500μl FACS buffer (0.5% BSA fraction V, 5mM EDTA, 1% Penicillin/Streptavidin, and 1μM thiazovivin in 1X PBS) and strained through a 30μm filter. Double-labeled cells were collected into 1 well of a 96-well plate by FACS. After 3 days, cells were collected and seeded at 1-$5x10^3$ cells in 10-cm plates. Individual colonies were then picked into 96-well plates.

To identify tetraploid colonies, we performed propidium iodide staining (Invitrogen, cat #P3566) on fixed cells to examine ploidy via FACS analysis. Briefly, cells were fixed in 80% EtOH overnight at 4°C. The next day, cells were washed twice in 1X PBS and stained at 37°C for 10 minutes in 20μg/ml RNase A, 40μg/ml of propidium iodide, and 0.1% Triton X-100 in 1X PBS. Cells with both 4N and 8N DNA content by FACS, suggesting that they contain tetraploid cells, were expanded. Expanded colonies were further screened for DNA content by karyotyping as described previously (4). Colonies that contained only tetraploid cells were maintained as stocks. G-banded karyotyping was also performed by WiCell (Dataset S1).

Trilineage differentiation. Diploid and tetraploid iPSC lines were seeded in 12-well plates and differentiated with the STEMdiff™ Trilineage Differentiation Kit according to the manufacturer's instructions (STEMCELL Technologies, cat #05230). Additionally, untreated cells were collected two days after seeding. Three replicate wells of cells per cell line were collected per condition. Differentiation was assessed using reverse transcription quantitative PCR (RT-qPCR) for pluripotency, ectoderm, mesoderm, and endoderm gene markers (Quantitative PCR SI Methods).

Quantitative PCR (qPCR). RT-qPCR was used to assess differentiation potential for trilineage differentiation samples, and qPCR was used to study DNA marker dosage in chrX targeted cell lines. All reactions were performed using Brilliant II SYBR Green Low ROX qPCR Master Mix (Agilent, cat #600830) on a QuantStudio 5 Real-Time PCR System (Thermo Fisher).

For trilineage differentiation, three replicate wells of cells per condition were collected in Trizol and applied to the Direct-Zol RNA Miniprep kit (Zymo Research, cat #R2051) for RNA extraction per the manufacturer's instructions. cDNA was synthesized from RNA with the SuperScript™ VILO™ cDNA Synthesis Kit (Thermo Fisher, cat #11754250). To assess differentiation potential of trilineage differentiation samples, qPCR was performed in triplicate on all samples with two marker genes each for pluripotency (*NANOG*, *DNMT3B*), ectoderm (*PAX6*, *RAX*), mesoderm (*TBXT*, *HAND1*), endoderm (*FOXA2*, *SOX17*), and housekeeping (*GAPDH*, *YWHAZ*) (5–11). All primer pairs span a large intron, have efficiencies between 90-110%, bind identical sequences in humans and chimpanzees, produce PCR products of identical length in both species, and were chosen from the literature or designed in-house (Dataset S2). For each sample, the quantity of each marker gene was calculated by comparing to a standard curve of pooled samples. This quantity was normalized by dividing by the geometric mean of the quantities of the two housekeeping genes (*GAPDH*, *YWHAZ*) in the same sample and then divided by the normalized quantity of the marker gene in undifferentiated iPSCs from the H2 human diploid line. Two-tailed Student's t-tests were used to determine statistically significant differences in marker gene expression between differentiated and undifferentiated iPSCs at 5% Benjamini-Hochberg FDR.

For determination of chrXq dosage relative to other chromosomes, cells were harvested from 96-well plates using Accutase (Millipore, cat #SCR005), and DNA was extracted using the DNeasy 96 Blood & Tissue Kit (Qiagen). Reactions were performed either in duplicate or in triplicate with primers for chromosomes 6p, Xp and Xq (Dataset S2).

Library preparation for RNA sequencing. Samples were flash frozen and stored at -80°C as a pellet. RNA extraction, library preparation, and sequencing for the chrX deletion samples were performed by Genewiz. All other RNA sequencing samples were prepared in-house before sequencing on the Illumina HiSeq 4000 with Novogene. Briefly, samples were resuspended in Trizol and directly applied to the Direct-Zol RNA Miniprep kit (Zymo Research, cat #R2051) for RNA extraction per the manufacturer's instructions. Technical replicates for each line were collected from thaws of different frozen vials. Only samples with $RIN > 9$ were used for RNA sequencing.

1μg of RNA was used for library preparation. RNA sequencing libraries were prepared with the TruSeq Stranded mRNA Library Prep (Illumina, cat #20020595) using the IDT for Illumina – TruSeq RNA UD Indexes (96 Indexes, 96 Samples) (Illumina, cat #20022371) according to the manufacturer's instructions with one modification. Prior to PCR amplification, 10% of a subset of samples were run under the recommended PCR conditions with SybrGreen on the QuantStudio 5 Real-Time PCR System. We identified the number of PCR cycles required to reach the crossing point by qPCR and used that number of cycles for PCR amplification on the entire set of samples. We ran 8 PCR cycles. Libraries were pooled and sequenced to around 10 million reads per sample for the diploid and auto-tetraploid lines and around 20 million reads per sample for the allo-tetraploid lines. Five independently-derived C1C1 auto-tetraploid lines, two independently-derived H1H1 auto-tetraploid lines (two technical replicates each), twelve independently-derived H1C1 allo-tetraploid lines, and ten independently-derived H2C2 allo-tetraploid lines were sequenced. Three technical replicates were sequenced for each diploid line.

Alignment of RNA sequencing to composite human-chimpanzee genome. Sequencing reads were trimmed for adapter sequences using cutadapt v1.8.1 (12), and read quality was confirmed using fastqc v0.11.9 (13). Reads were aligned using STAR v2.7.1a with two-pass mapping (14). Samples were mapped to a composite human-chimpanzee genome (hg38 and pt6). The number of uniquely-mapped reads ($MAPQ = 255$) that overlap each gene was counted using featureCounts from the subread v1.6.0 package (15).

To generate the gene annotations used in featureCounts, GRCh38.94 human exon annotations from Ensembl (16) were mapped from hg38 to pt6 using pslMap (17). After removing mappings where the number of bases that map is less than half of the query exon size, we retained only exons that uniquely mapped from humans to chimpanzees. We then removed genes for which exons map to opposite DNA strands, different scaffolded chromosomes, or where consecutive exons map more than 800kb apart. We further filtered out exons where more than 10% of reads from diploid or auto-tetraploid lines map to the incorrect species when mapped to the composite genome. This resulted in 48,735 annotated genes that contain at least 1 exon (byexon-gene). We also used a second set of annotations. We identified SNPs that differed between the human and chimpanzee cell lines using the GATK RNA variant pipeline (18, 19) and assigned SNPs to genes annotated in humans. We also filtered out SNPs where more than 10% of reads from diploid or auto-tetraploid lines map to the incorrect species when mapped to the composite genome. This resulted in 14,333 annotated genes with at least 1 SNP (bysnp-gene). Read counts for the byexon-gene annotation were also adjusted for feature length to account for differences between feature length in the human and chimpanzee genomes. Results were very similar across both annotations, and results are reported for the byexon-gene annotation in the current study.

Gene expression analysis of diploids and auto-tetraploid iPSC lines. After sequencing reads were aligned to the composite human-chimpanzee genome as described above, differential gene expression analysis was performed with DESeq2 (20) using default parameters. We called genes as significant if they had an adjusted $p < 0.05$ after Benjamini-Hochberg FDR correction and at least a 2-fold change in expression.

Differential gene expression, allele-specific gene expression, and *cis*/*trans* analysis between humans and chimpanzees in diploid, auto-tetraploid, and allo-tetraploid iPSC lines. Differential expression (DE) between single-species iPSCs, allele-specific expression (ASE) in allo-tetraploids, and regulatory type classifications were carried out as a combination of previously described methods (21, 22).

After RNA sequencing reads were aligned to the composite human-chimpanzee genome as described above, reads mapping to genes on human chromosome 18 (and the orthologous chimpanzee genes) were removed. Next, each sample was downsampled to 9,711,244 reads (for DE) or 11,490,119 reads (for ASE), and genes with fewer than 10 reads assigned to both the human and the chimpanzee orthologs were excluded. For each gene, $log_2(FC)$ was calculated between each human-only and each chimpanzee-only sample (for DE) or between the human allele and the chimp allele in each allo-tetraploid cell line (for ASE). Genes with significantly different $log_2(FC)$ between human and chimpanzee were determined to be DE or ASE. Each gene was tested for significant "*trans*-effects" by testing for a significant $log_2(FC)$ difference between single-species iPSCs and allo-tetraploid iPSCs. Significance for all $log_2(FC)$ differences was determined by Welch's t-test at 5% Benjamini-Hochberg FDR . Importantly, only half of the allo-tetraploid samples were used to determine whether a gene is significantly ASE, and the other half were used to determine significant "*trans*-effects" since this has been reported to reduce false classification as *compensatory* (23).

Finally, the *cis*-contribution (C) and *trans*-contribution (T) to the observed DE $log_2(FC)$ (D) was calculated for each gene. Specifically, the *cis*-contribution (C) was equal to the ASE $log_2(FC)$, and the *trans*-contribution was calculated as $T = D - C$.

Genes were classified by regulatory type based on the following criteria:

cis: significant DE, significant ASE, no significant "*trans*-effects," *cis*-contribution and *trans*-contribution to DE $log_2(FC)$ in the same direction

trans: significant DE, not significant ASE, significant "*trans*-effects"

cis+trans: significant DE, significant ASE, significant "*trans*-effects," *cis*-contribution and *trans*-contribution to DE $log_2(FC)$ in the same direction

cis-trans: significant DE, significant ASE, significant "*trans*-effects," *cis*-contribution and *trans*-contribution to DE $log_2(FC)$ in opposite directions

compensatory: not significant DE, significant ASE, significant "*trans*-effects"

conserved: not significant DE, not significant ASE, no significant "*trans*-effects"

ambiguous: all other patterns

All results reported in this paper used the "by-exon-gene" annotation as described in the "Alignment of RNA sequencing to composite human-chimpanzee genome" section above (except for *TSPAN6*, which was not included in the "byexon-gene" annotation and was assessed using the "bysnp-gene" annotation).

Gene ontology enrichments. Significant gene ontology enrichments (adjusted $p < 0.05$ after Benjamini-Hochberg FDR correction) were determined using the R package clusterProfiler's enrichGO function (24) for the annotation data sets "Biological Process," "Molecular Function," and "Cellular Component." The set of analyzed genes was used as the background reference list.

Gene expression analysis of X chromosome deletion lines. RNA sequencing reads were aligned to the composite human-chimpanzee genome as described above. To identify the approximate location of X chromosome deletions, we computed the ratio of human read counts to chimpanzee read counts for each deletion line normalized to control (non-deletion) lines. A count of 1 was added to any sample with allele counts of zero, and ratios were calculated for genes with more than 10 counts on average and where at least half of the samples had at least 5 counts. Approximate deletion breakpoints were then determined by visual inspection.

To identify autosomal genes whose expression may be affected by *trans*-regulators on the X chromosome, we carried out differential gene expression analysis of control and human and chimpanzee chrX targeted deletion lines using DESeq2 (20) with the Wald test at 5% Benjamini-Hochberg FDR. *Trans*-regulated candidates were identified by the following five criteria: (1) Genes on autosomes that showed significant expression changes when comparing the four lines with deletion breakpoints of the chimpanzee chrX around 148Mb (cXdel4-cXdel7) to the nine control lines that lack deletions; (2) Genes on autosomes that did not show significant expression changes when comparing the five lines with deletion breakpoints of the human chrX around 95Mb (hXdel3-hXdel7) to the nine control lines that lack deletions; (3) Genes that met the first two criteria whose expression level was also significantly different in comparisons between the chimpanzee (cXdel4-cXdel7) and human (hXdel3-hXdel7) terminal deletions; (4) Genes that also showed the same direction of change in cell lines carrying shorter (cXdel4-cXdel7) and larger chimpanzee chrX deletions (cXdel1-cXdel3) compared to control lines; and (5) Genes where the hXdel8 line which has a human deletion breakpoint near the distal chimpanzee chrX deletion lines maintained expression within the range of the control lines.

Sister chromatid exchange (SCE) assay. Cells were passaged the day before testing. For camptothecin (Sigma Aldrich, cat #C9911-100MG), camptothecin and 10μM BrdU were applied to cells for 1 hour before being replaced with fresh media containing 10μM BrdU overnight. For ML216 (Cayman Chemical, cat #15186) and mitomycin C (Sigma Aldrich, cat #M4287-2MG), cells were incubated with the small molecule and 10μM BrdU for 24-48 hours with a media change every 24 hours. Cells were then moved to fresh media containing 10μM BrdU and 0.1μg/ml colcemid for 4 hours and subsequently collected for sister chromatid exchange (SCE) assay as previously described (25). Cells were alternatively first collected into a 1.5ml tube before adding new media containing 10μM BrdU and 0.1μg/ml colcemid, with no obvious change in results. Multiple metaphase spreads were imaged at 100X, and recombination events were counted using the ImageJ Cell Counter function. *P*-values were calculated using the 1-tailed Student's t-test.

Haplotagging. Haplotagging was performed as previously described (26). Briefly, genomic DNA from each sample was mixed with individually barcoded magnetic beads containing bead-immobilized active Tn5 transposase for tagmentation with up to 21 million barcode diversity. Tagged DNA was then PCR amplified, size selected, and sequenced on a NovaSeq 6000 instrument (Illumina).

Reads were aligned to a composite human-chimpanzee genome (hg38 and pt6) using EMA, a barcode-first variant of the bwa aligner (27). For the analysis, we focused on regions that reciprocally and uniquely mapped between the two species assemblies, with the mapping based on the hg38 to pt6 chain files from the UCSC Genome Browser (28) and pslMap (17). 500bp orthologous regions with greater than 2-fold difference in read coverage were excluded from further analysis. Each read was also assigned to a molecule based on its barcode (retained as the BX beadTag). For each read, we identified variants

between hg38 and pt6 (SNPs and indels). The variant annotation file was generated by first parsing the maf file between hg38 and pt6 from the UCSC Genome Browser (28). We also included variants identified by running the GATK variant pipeline (18, 19) on reads that map uniquely to either hg38 or pt6 and where all reads assigned to a given barcode map to only one species. If no variants in our resulting annotation file were identified in a read but the read uniquely mapped to either hg38 or pt6, the read itself was considered as a variant.

Along each molecule, we coded the species assignment (e.g H-H-H-H-H-C-C-C where H is a human variant and C is a chimpanzee variant). We then applied the following strict filters to identify a high-confidence set of recombinant molecules: (1) Identified SNPs must have a phred quality score of at least 30; (2) Given the low rate of mitotic recombination, multiple "switch" events (e.g. H-H-H-C-H-H-H) are likely artifacts and such variants were removed; (3) We also excluded possible mapping artifacts where particular variants were found at the boundary of multiple recombination events; (4) Variants contained in 500bp regions with greater than 2-fold difference in the directionality of switch events (e.g. switch events were predominantly $H \rightarrow C$ instead of $C \rightarrow H$) were removed; (5) We included only paired recombinant molecules that could be "reciprocal events" to further account for biases in the directionality of switch events; (6) We excluded any variants that are in ENCODE blacklist regions (29); (7) All recombinant molecules must contain only 1 switch event and > 5 supporting variants per species.

To calculate the genome-wide recombination rate, we divided the number of recombination events by the approximate number of analyzed human-chimpanzee tetraploid genomes (molecular coverage of molecules that passed the above filters). To compare the inter-specific recombination rate from haplotagging to previously reported recombination rates in the literature, we examined previous reports that selected for recombination events near single-locus, drug-selectable markers following ML216 treatment in human or mouse embryonic stem cells (30–32). To extrapolate single-locus marker rates to genome-wide estimates, we calculated the genomic distance between the centromere and the drug-selectable marker, and estimated the genome-wide recombination rate as (size of diploid genome / genomic distance studied) * reported recombination rate.

To assess the effect of CRISPR targeting to specific loci, we examined the recombination rate in the 250kb interval surrounding the target loci with and without filters, with no difference in the relative enrichment at the target loci. Data visualizations were generated with ggplot2 (33) and karyoploteR (34). In Fig. S6, samples were plotted with their experimental batch due to differences in read and molecular coverage.

Generation of fluorescently-tagged allo-tetraploid lines. We cloned two plasmids, one with homology arms (chrX:153,850,316-153,851,493, hg38) flanking a EF1a-EGFP-IRES-PuroR cassette to target human chrX and the second with homology arms (chrX:149,205,726-149,208,867, pt6) flanking a EF1a-mCherry-IRES-NeoR cassette to target chimpanzee chrX (Fig. S7), into the pMAXGFP plasmid backbone (Lonza). Guide RNAs (gRNAs) were designed to linearize the plasmid containing the insertion cassette and cut the target insertion site (HR_X_gRNA_1 and HR_X_gRNA_2 in Dataset S2). gRNAs were then *in vitro* transcribed as described above.

2.5μl of 40μM Cas9-NLS purified protein (QB3, UC Berkeley) was mixed with 2.5μg each of both gRNAs for 10 minutes at room temperature. This complex and 1.875μg of the plasmid targeting human chrX were nucleofected into $3x10^6$ cells using the Nucleofection Stem Cell Kit 2 (Lonza, cat #VPH-5022) and program A-33 on the Nucleofector 2b Device (Lonza). Immediately after nucleofection, 1ml of pre-warmed media (mTeSR1 + 1μM thiazovivin) was added to the reaction. The reaction was allowed to recover for 20 minutes at room temperature, and 5 separate reactions were pooled and plated on one 10-cm plate. We also nucleofected pMAXGFP separately as a positive control for nucleofection efficiency.

After cells recovered and expanded (~5 days post-nucleofection), cells with insertion events were selected by multiple days of puromycin treatment. We examined selection efficacy via fluorescence under an EVOS FL microscope. After multi-day selection, we picked colonies into 96-well plates. When colonies reached confluency, they were split and screened for proper insertion events by PCR, using primer pairs where one primer targets nearby genomic DNA and a second primer targets the insertion construct. We verified target-site insertion events using primer sets at both the 5' and 3' ends and separately with species-specific primers (Dataset S2). We confirmed the insertion sequence via PCR followed by Sanger sequencing with primers chrX-F2 and chrX-R2 (Dataset S2). To confirm that the insertion was inserted into the target locus and nowhere else in the genome, we expanded promising colonies for Southern blot analysis. Colonies verified by both PCR and Southern blot were then subject to a second round of nucleofection to insert the mCherry cassette into chimpanzee chrX. Double-marked colonies were selected for using Geneticin (Thermo Fisher, cat #10131035) and puromycin (Sigma-Aldrich cat #P8833). Double-marked lines were confirmed by PCR, Southern blot, and visual inspection of fluorescence.

CRISPR/Cas9 treatment of iPSC lines. Guide RNAs were designed (Dataset S2) and *in vitro* transcribed (IVT) as previously described (35). Briefly, CRISPR IVT target oligos containing the gRNA and the CRISPR IVT scaffold oligo (HPLC-purified) were synthesized by Integrated DNA Technologies. 40 cycles of PCR were performed between the CRISPR IVT scaffold oligo using Phusion DNA polymerase (Thermo Scientific, cat #F530L) and the CRISPR IVT target oligo, and the PCR product was purified using the QIAquick PCR Purification Kit (Qiagen, cat #28104). The PCR product was then *in vitro* transcribed using the MEGAscript T7 transcription kit (Thermo Fisher, cat #AM1334) for 16 hours at 37°C. The reaction was treated with DNase, and transcribed gRNA was extracted with phenol/chloroform and precipitated with isopropanol. Transcribed gRNA was resuspended to approximately 2μg/ul.

To select the highest efficiency guides, we tested performance in 96-well plate format. For each guide, 1μl of 40μM Cas9-NLS purified protein (QB3, UC Berkeley) was complexed with 2μg of gRNA for 10 minutes at room temperature. We performed nucleofection of $2x10^5$ cells per reaction using the P3 Primary Cell 96-well Nucleofector Kit (Lonza, cat #V4SP-3096) on the Amaxa 96-well Shuttle Device (Lonza) with program CA-137. Two days post-nucleofection, cells were collected for DNA

extraction using phenol/chloroform. We used primers bracketing the target cut site (Dataset S2) and Sanger sequenced the products for analysis using TIDE (36) to determine guide efficiency. For a subset of guides, we further confirmed cutting events by cloning the gel-extracted PCR product into the TOPO TA vector (Life Technologies, cat #450641) and performing colony PCR followed by Sanger sequencing to identify lesions at the target cut site.

For targeted recombination, we nucleofected cells with CRISPR/Cas9 and gRNA using the same nucleofection conditions as described above in the "Generation of fluorescently-tagged allo-tetraploid lines" section. For CRISPR+ML216 conditions, we treated cells with 25μM ML216 starting 12 hours before nucleofection, as previously described (37). After recovering for 1 hour, nucleofected cells were plated directly into media with ML216, and ML216 media was replaced again after 24 hours. At 48 hours post-nucleofection, cells were collected for FACS or haplotagging experiments.

Fluorescence activated cell sorting (FACS). Allo-tetraploid cells with fluorescently-marked chrX were subjected to CRISPR+ ML216 treatment as described above.

For cells treated with chimpanzee-specific gRNA, we selected for loss of mCherry (from the chimpanzee chrX insertion) and possible duplication of GFP (from the human chrX insertion) by sorting for mCherry-negative, high-intensity-GFP cells. To eliminate cells with high GFP due to duplicated DNA content at the G2/M phase of the cell cycle, we stained cells with Hoechst 33342 (Thermo Fisher, cat #62249) for 30 minutes at 37°C to sort only from cells in the G1 cell cycle phase.

For cells treated with human-specific gRNA, we selected for loss of GFP (from the human chrX insertion) and possible duplication of mCherry (from the chimpanzee chrX insertion) by sorting for GFP-negative, high-intensity-mCherry cells. As a second marker of two copies of chimpanzee chrX downstream of the gRNA cutsite, we chose a cell-surface protein, TSPAN6, that has *cis*-regulatory changes with 1.4-fold higher expression in chimpanzee relative to human (adjusted $p = 1.4x10^{-4}$ by Welch's t-test after Benjamini-Hochberg FDR correction). Because this human-chimpanzee gene expression difference can also be observed at the level of protein expression by antibody staining, sorting for high TSPAN6 protein acted as a second marker to potentially sort for two copies of chimpanzee chrX downstream of the gRNA cutsite (Fig. S9).

Treated cells were stained with either Hoechst 33342 at 10μg/mL for 30 minutes at 37°C, or with TSPAN6 primary antibody (1:10; LS Bio, cat #LS-C160272-400) for 1 hour at 4°C followed by 30 minutes at 4°C with a goat anti-rabbit secondary antibody conjugated with APC fluorophore (1:500; Thermo Fisher, cat #A-10931). Cells were sorted single-cell into 96-well plates on a BD Influx cell sorter at the Stanford Shared FACS Facility. Representative sorting gating schemes are shown in Fig. S9.

DNA sequencing analysis of chrX recombinant and deletion lines. DNA from the recombinant allo-tetraploid cell line (H1C1a-X1-Xrec1), two chimpanzee chrX deletion lines (H1C1a-X1-cXdel5 and H1C1a-X1-cXdel6), and a control allo-tetraploid line (H1C1a-X1-S) (Dataset S1) were extracted and sequenced to 30X coverage with 150bp paired-end reads by GeneWiz. Illumina adapters were removed using Picard Tools (http://broadinstitute.github.io/picard/), and reads were then aligned to a composite human-chimpanzee (hg38-pt6) reference genome using BWA-MEM with the -M flag (38). Duplicate reads were marked with Picard Tools and removed using samtools (39). We filtered out reads with $MAPQ < 30$ and reads that did not lift over between hg38 and pt6 using pslMap (17). For the recombinant line (Fig. S10) or the deletion lines (Fig. S12), we normalized observed read counts to the read counts in the control H1C1a-X1-S over 10kb sliding windows to account for any sequencing or mapping bias and visualized this ratio along human chrX coordinates. For the recombinant line, inspection of reads at the likely recombination site revealed the exact junction site as a 4bp microhomology (CACC) found at both human chrX:140133478-140133481 (hg38) and chimpanzee chrX:124020937-124020940 (pt6).

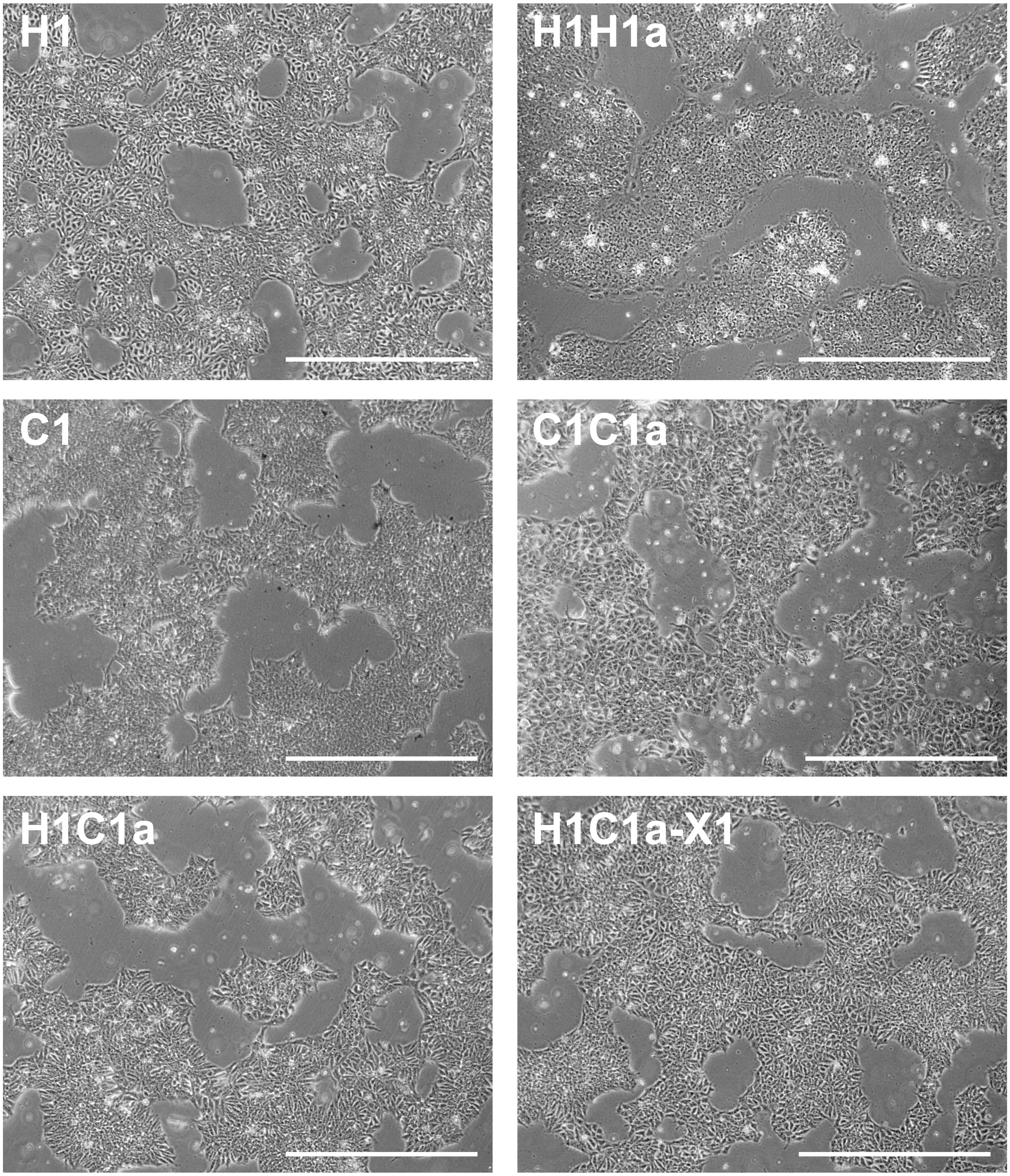

Fig. S1. Morphologies of auto- and allo-tetraploid iPSC lines are similar to those of diploid iPSC lines. Representative brightfield images of human diploid (H1), human auto-tetraploid (H1H1a), chimpanzee diploid (C1), chimpanzee auto-tetraploid (C1C1a), allo-tetraploid (H1C1a), and chrX-marked allo-tetraploid (H1C1a-X1) lines are shown. Scale bars are 1mm.

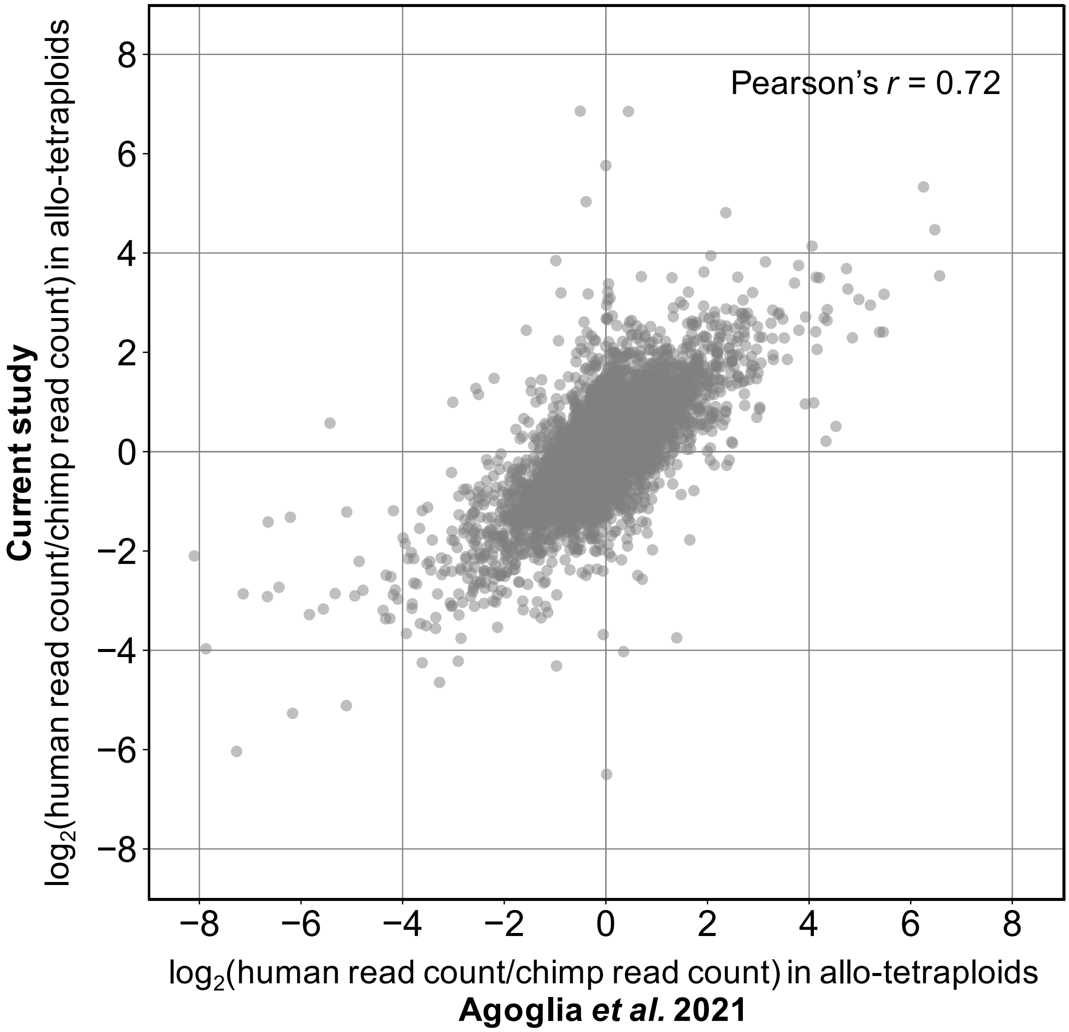

Fig. S2. Allele-specific expression in human-chimpanzee allo-tetraploid iPSCs is reproducible across studies. Allele-specific expression (ASE) $log_2(FC)$ values from RNAseq generated by Agoglia *et al.* 2021 (40) (x-axis) and ASE $log_2(FC)$ values from the RNAseq data reported in this study (y-axis) are highly concordant (Pearson's $r = 0.72$). Allo-tetraploid cells were derived from independent human-chimpanzee iPSC fusion events in the two studies, and different pipelines were used for mapping reads, assigning reads to the human or chimpanzee version of a gene, and calling genes with significant ASE. ASE differences in human-chimpanzee allo-tetraploid iPSCs are thus highly reproducible and robust to different analysis methods.

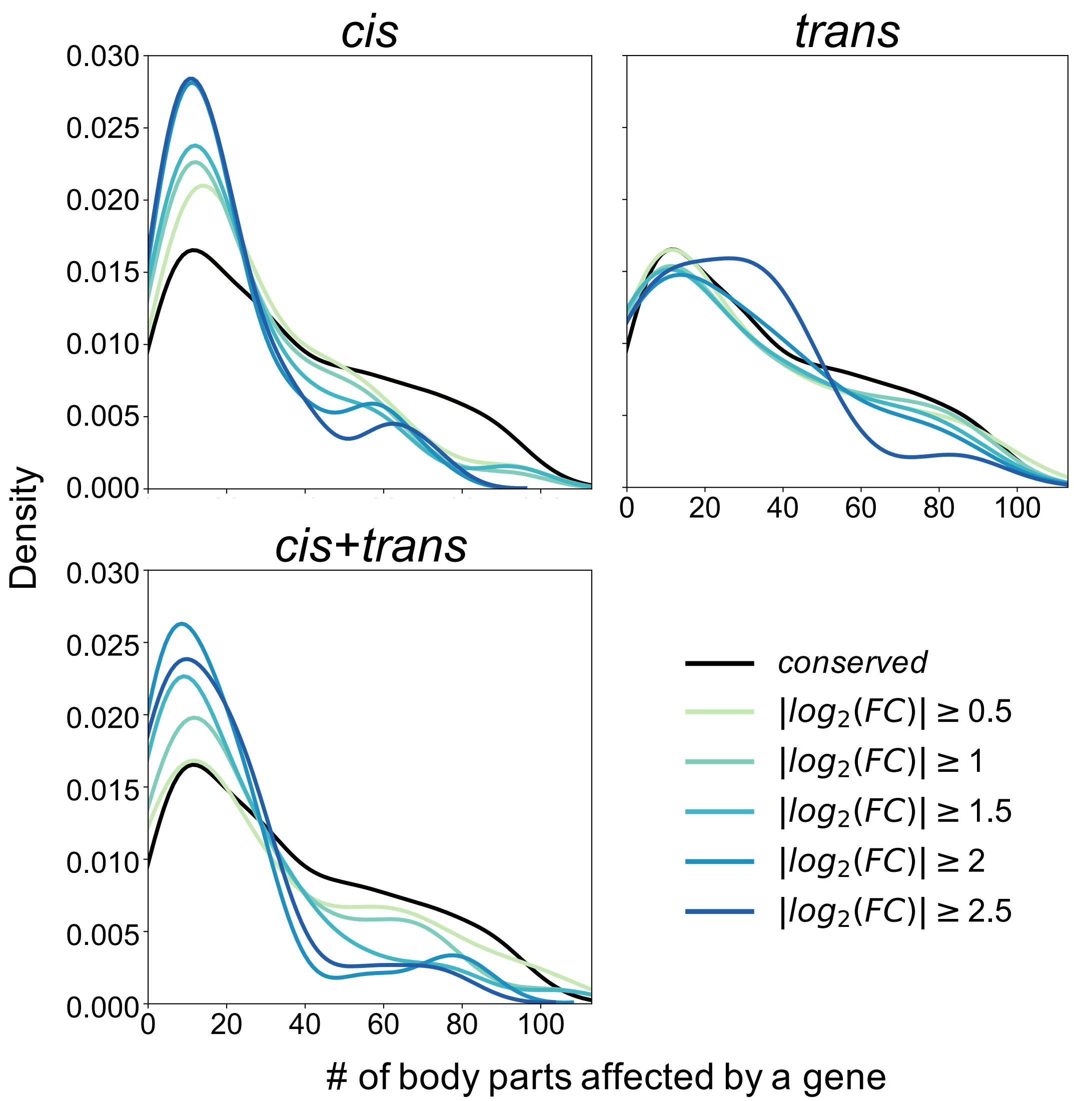

Fig. S3. Genes with increasingly divergent expression between human and chimpanzee iPSCs influence fewer body parts for *cis* and *cis+trans* regulatory types. Density plots (smoothed histograms) showing the distribution of body parts influenced by genes (according to the Gene ORGANizer database (41)) with human-chimpanzee expression differences due to *cis* (upper left), *trans* (upper right), and *cis+trans* (lower left) regulatory changes at increasing $|log_2(FC)|$ cutoffs. The *cis-trans* category is not included because only 5 genes have $|log_2(FC)\geq 1|$. For genes classified as *cis* and *cis+trans*, the median number of body parts influenced decreases with higher $|log_2(FC)|$ cutoffs (22, 18, 17, 15, 15 body parts and 22.5, 18, 16, 14, 14 body parts, respectively, for $|log_2(FC)|\geq 0.5, 1, 1.5, 2, 2.5$). All comparisons between the median number of body parts influenced by *conserved* genes (median of 30 body parts influenced) and by *cis* or *cis+trans* genes at the various $|log_2(FC)|$ cutoffs are statistically significant (adjusted $p < 0.04$ by two-tailed Mann-Whitney U test after FDR correction). This trend does not hold for gene expression differences due to *trans*-regulatory changes (adjusted $p > 0.19$ for all comparisons between *conserved* genes and *trans* genes at the various $|log_2(FC)|$ cutoffs).

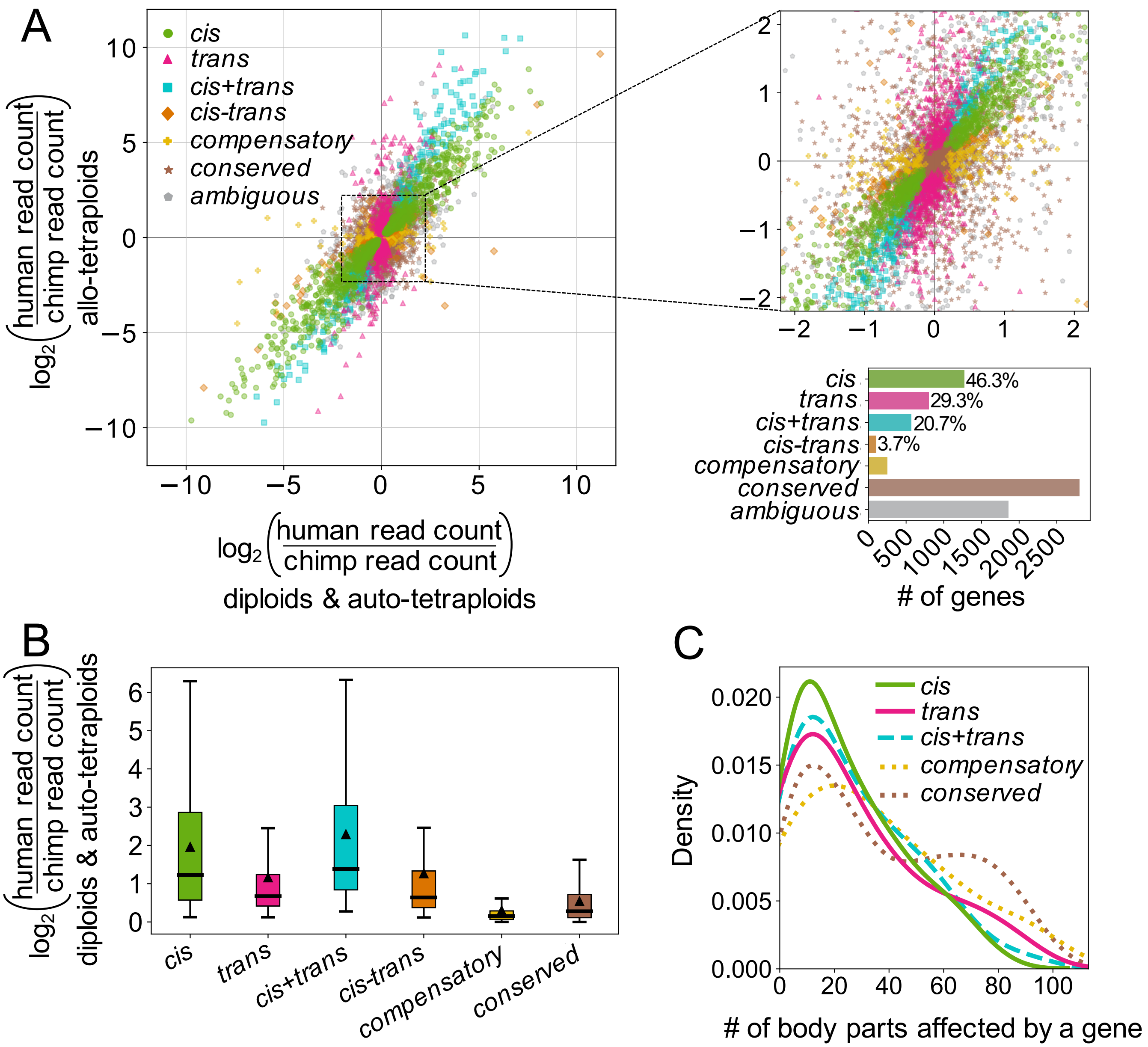

Fig. S4. ***Cis*** **and** ***trans*** **analysis results are robust to aneuploidies.** Removing chromosomes with aneuploidies or abnormalities in any of the cell lines used for RNAseq does not meaningfully change the observed *cis* and *trans* trends demonstrated in Fig. 2C-D. In addition to genes on human chromosome 18 and their orthologous genes in chimpanzee (deletion of one copy of human chr18q is shared by a subset of cell lines and was removed for the *cis* and *trans* analysis shown in Fig. 2C-E), genes on human chromosomes 7, 12, 20, and Y (and orthologous genes in chimpanzee) and chimpanzee chromosomes 1, 2A, 2B, 11, 13, 14, 17, 19, 20, and Y (and orthologous genes in human) were removed prior to analysis. **(A)** See Fig. 2C legend. **(B)** See Fig. 2D legend. All pairwise comparisons are statistically significant by two-tailed Mann-Whitney U test after FDR correction with adjusted $p < 10^{-5}$ except *trans* compared to *cis-trans* ($p = 0.28$). **(C)** See Fig. 2E legend. Genes classified as *cis*, *trans*, or *cis+trans* tend to influence fewer body parts than *conserved* genes (median 19, 20, 19.5 body parts, respectively, compared to median 29 body parts for *conserved* genes, adjusted $p = 0.0029, 0.024, 0.024$ by two-tailed Mann-Whitney U test after FDR correction). Note that the comparison between the *trans* and *conserved* categories is not statistically significant in Fig. 2E.

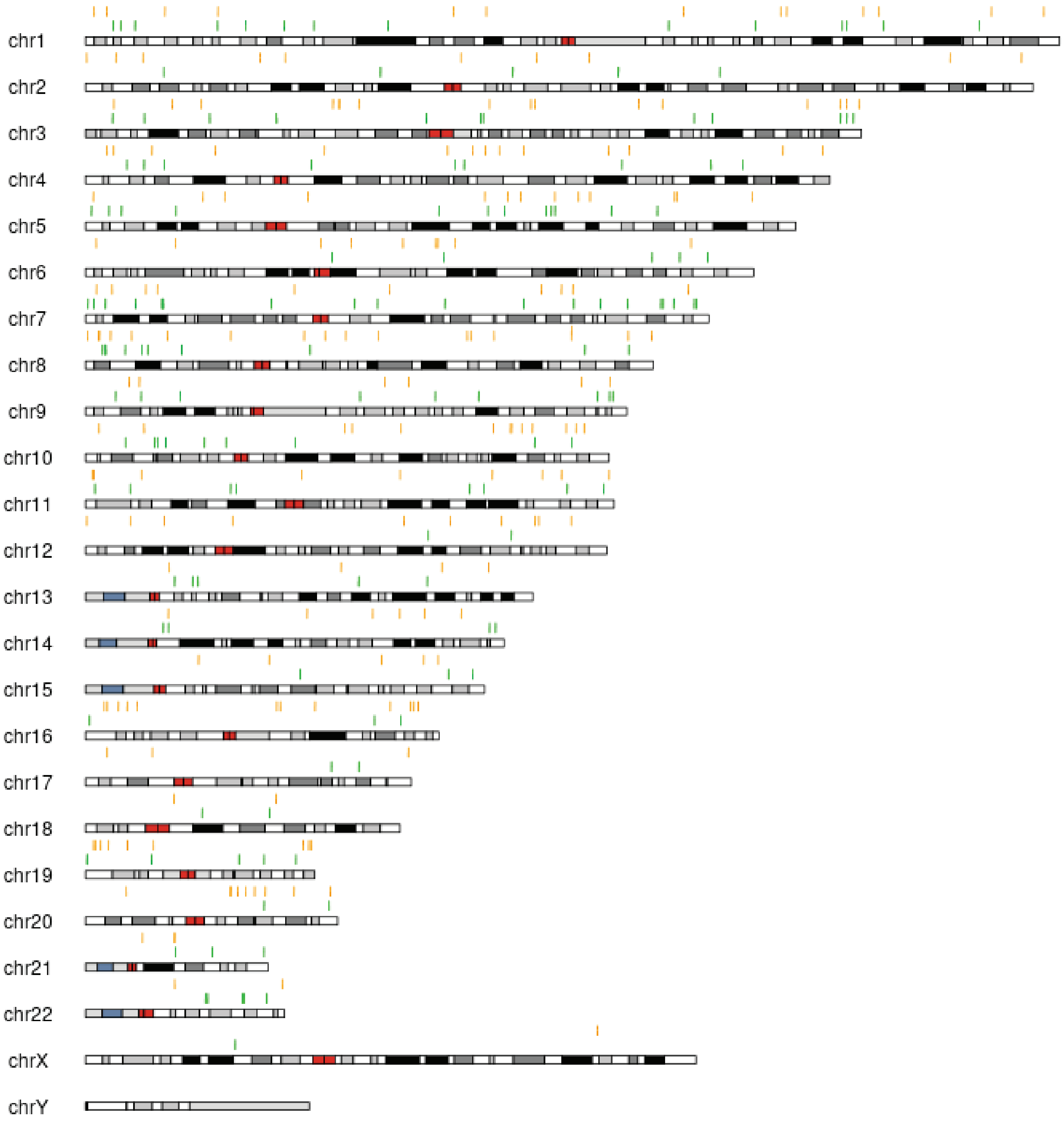

Fig. S5. Distribution of genome-wide inter-specific recombination events identified by haplotagging. Representative chromosome plots showing locations of inter-specific molecules detected by haplotagging after genome-wide sequencing and filtering. Green: cells treated with gRNA-chr20 (g20). Orange: cells treated with gRNA-chr20 and ML216 (g20+ML216).

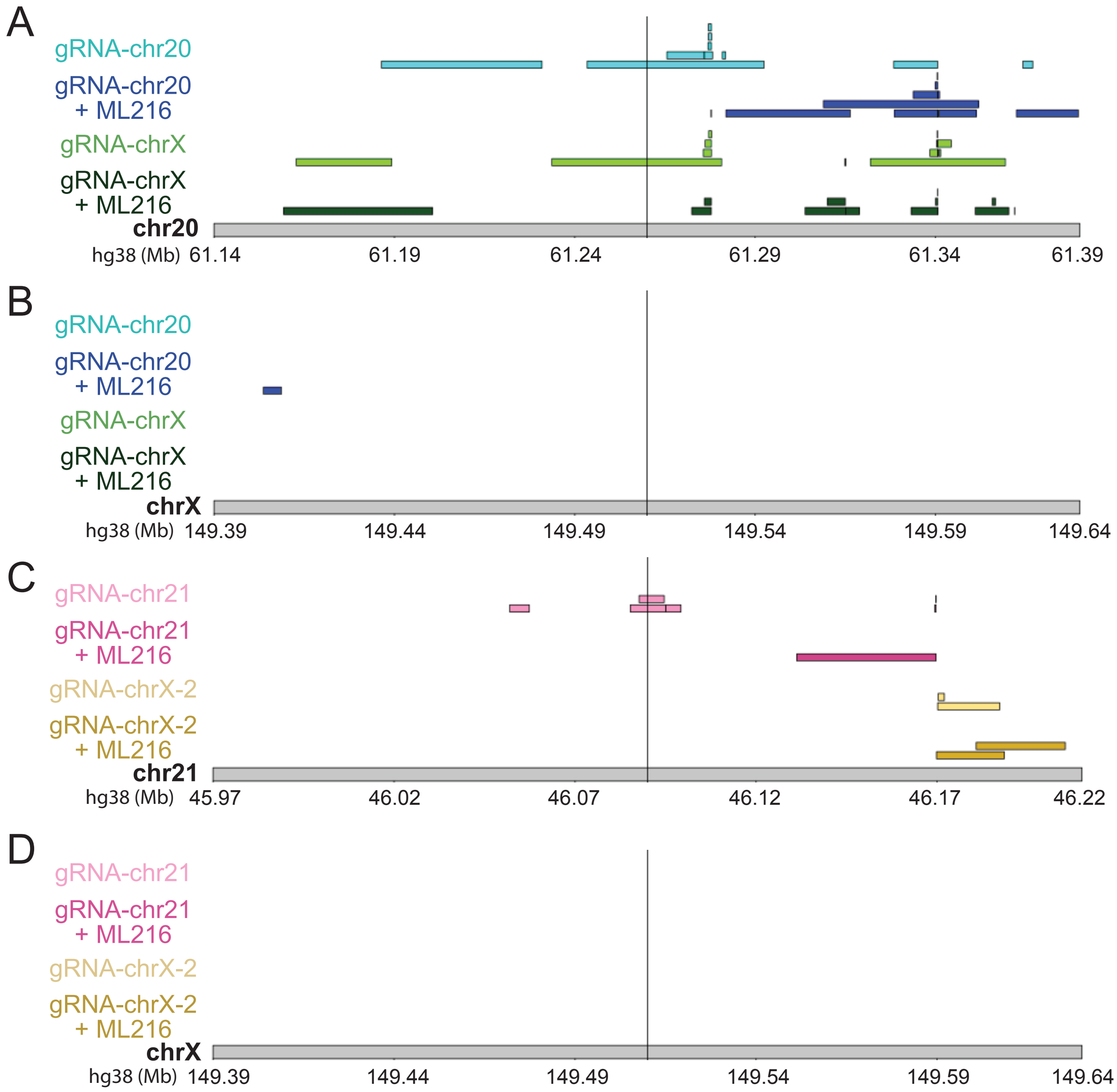

Fig. S6. CRISPR targeting does not elevate inter-specific recombination rates at target loci. Plot of inter-specific recombination events in the 250 kb window surrounding CRISPR target loci on chr20 **(A)**, chrX **(B)**, chr21 **(C)**, or a second guide location on chrX **(D)**. Each horizontal rectangle represents the boundaries of an inter-specific recombination event detected by haplotagging. Vertical lines indicate the gRNA target site. Events are filtered for molecules that contain only 1 inter-specific event and have > 5 supporting variants per species but are otherwise pre-filtering. The lack of enrichment at the target sites does not change with different filters (SI Methods).

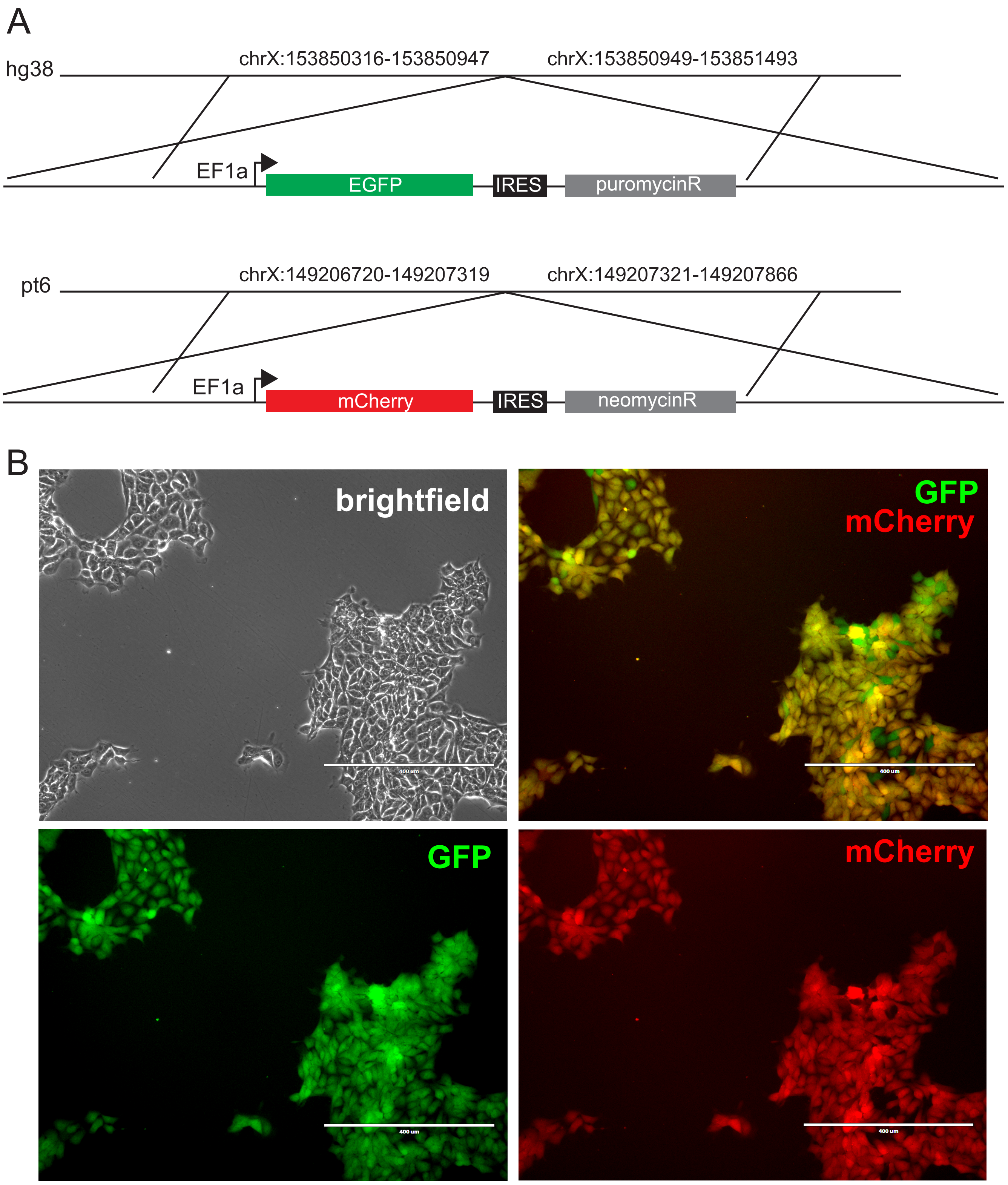

Fig. S7. Generation of fluorescently-marked allo-tetraploid lines. Construct diagram and microscopy images for fluorescently-marked line. **(A)** Constructs containing EGFP or mCherry were inserted onto the human or chimpanzee chrX, respectively, using CRISPR-guided homologous recombination (Materials and Methods). Coordinates show locations of human and chimpanzee homology arms used in the constructs. **(B)** Allo-tetraploid H1C1a-X1 shown in brightfield, GFP, and mCherry. Cells marked with both GFP and mCherry appear yellow.

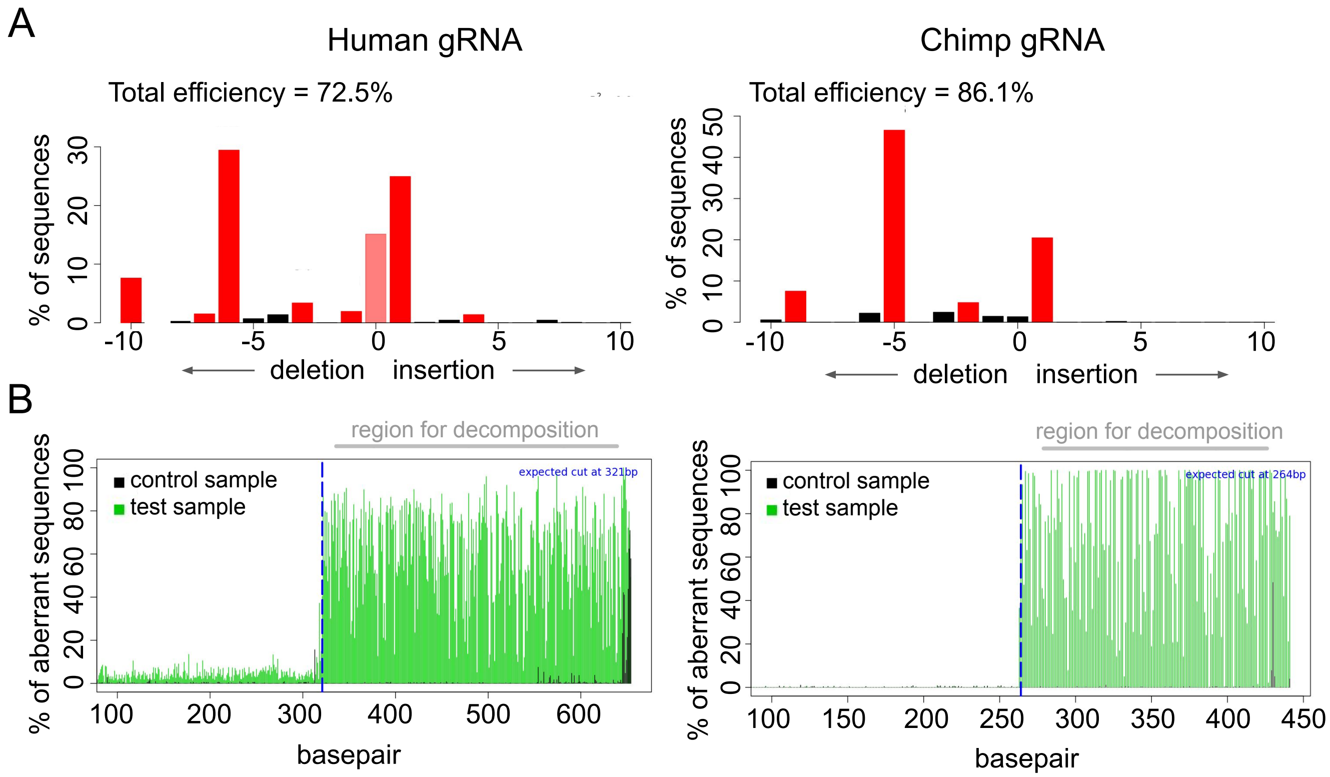

Fig. S8. CRISPR/Cas9 gRNA editing efficiency and indel spectra for human and chimpanzee chrX guides. (A) For human- and chimpanzee-specific gRNAs, the spectrum and frequency of small insertions and deletions, gRNA efficiency, and **(B)** aberrant sequence signal plots are shown. Plots generated with Sanger sequence data in TIDE (Tracking of Indels by DEcomposition) (36).

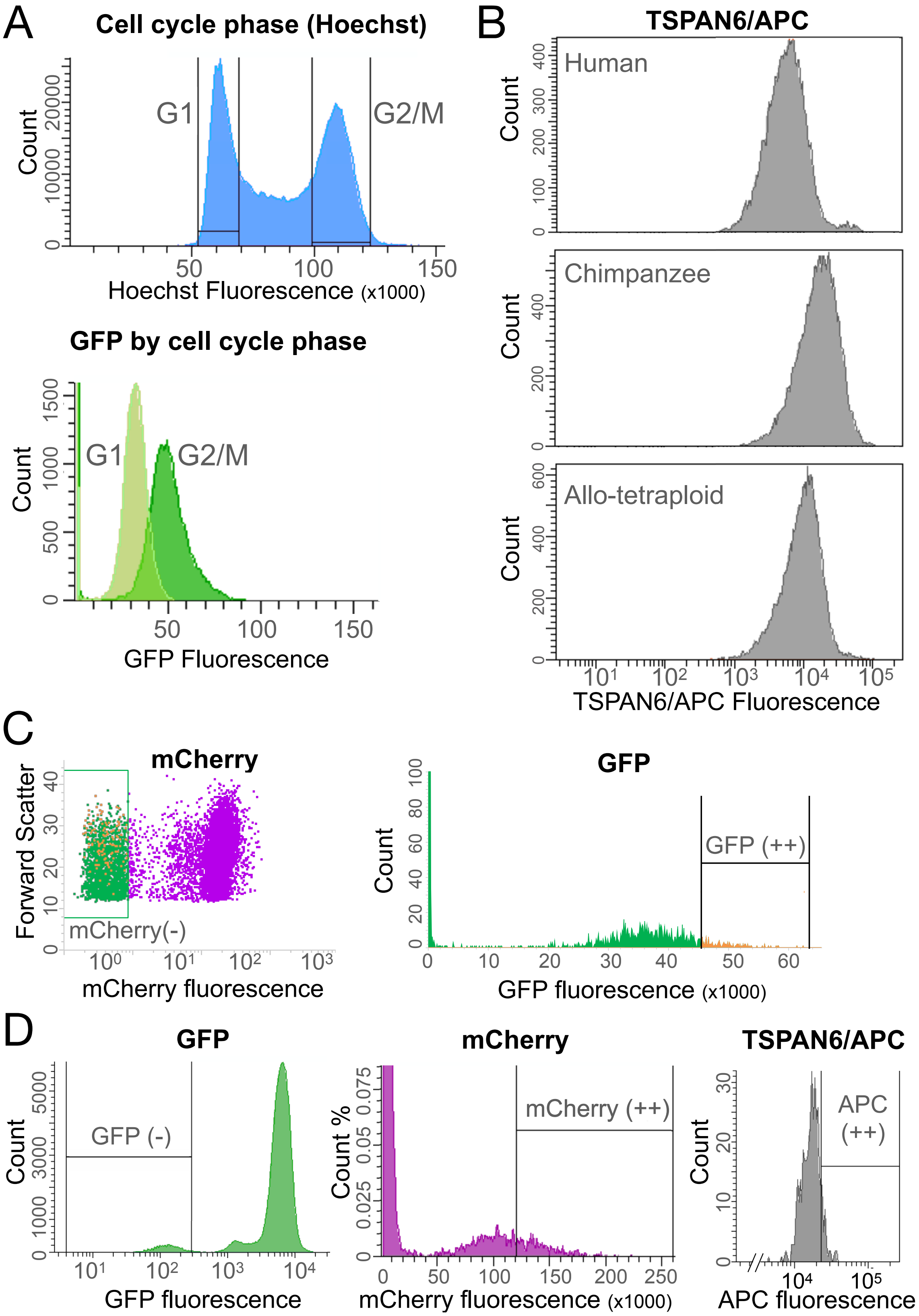

Fig. S9. Fluorescence activated cell sorting (FACS) plots for chrX targeting. (A) Cell cycle phase determined by Hoechst peaks shows that G2/M cells exhibit higher GFP fluorescence than G1 cells. **(B)** Staining for TSPAN6 cell-surface protein with APC secondary antibody shows that chimpanzee TSPAN6-APC fluorescence intensity is higher than human TSPAN6-APC fluorescence intensity, with allo-tetraploid cells intermediate between human and chimpanzee values. **(C)** After G1 gating, cells treated with chimpanzee-specific gRNA are sorted for negative mCherry and high GFP fluorescence. **(D)** Cells treated with human-specific gRNA are sorted for negative GFP, high mCherry, and high TSPAN6-APC.

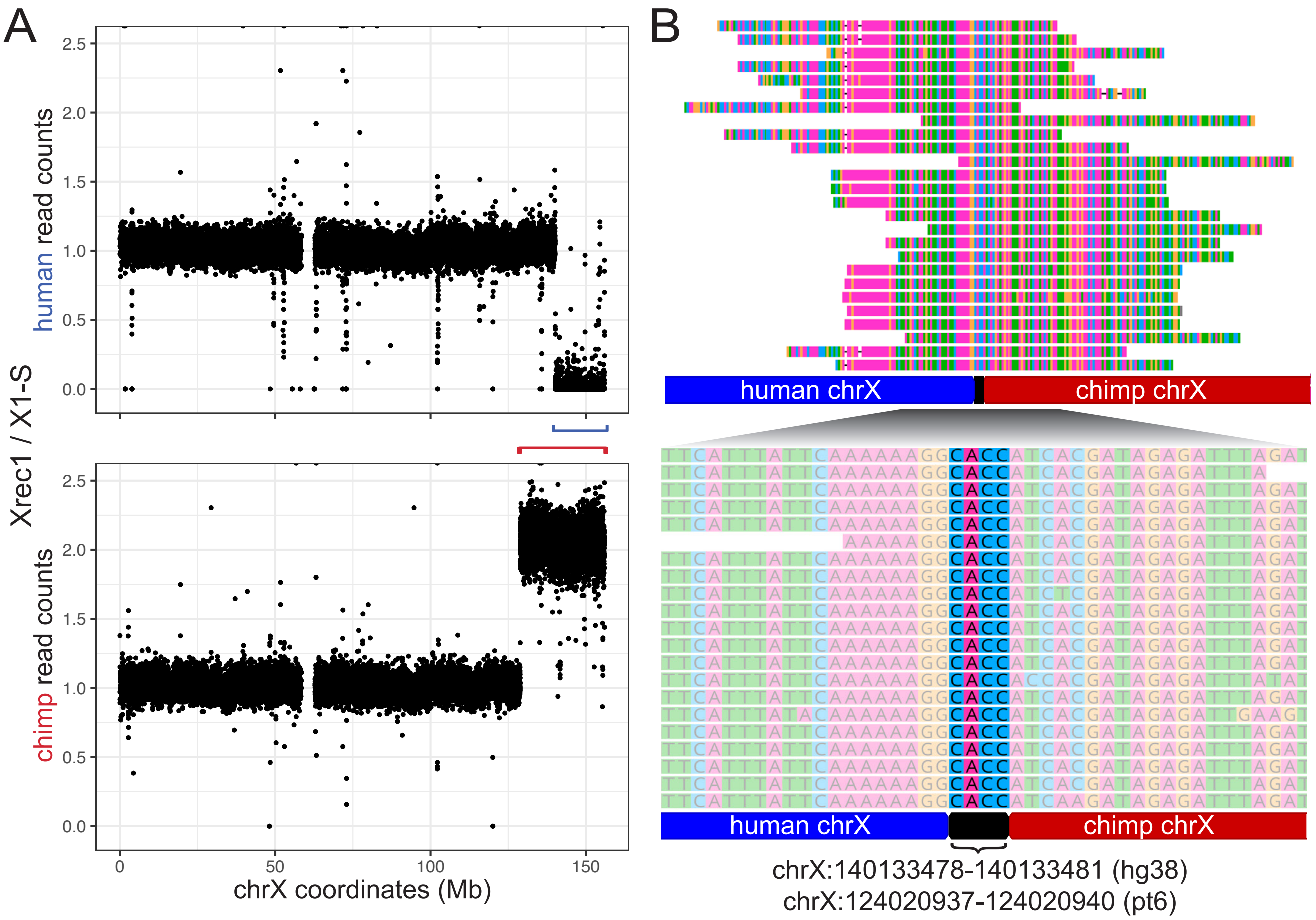

Fig. S10. DNA sequencing identifies the site of recombination between human and chimpanzee X chromosomes. Whole-genome DNA sequencing data from the recombinant allo-tetraploid line H1C1a-X1-Xrec1 (Xrec1). **(A)** Read counts that align to either the human or chimpanzee allele along chrX were normalized to read counts for H1C1a-X1-S (X1-S), a control sample also sequenced in parallel (see Materials and Methods). This ratio was plotted along the X chromosome in hg38 coordinates. Blue bracket: region with no human read counts in Xrec1. Red bracket: larger region with twice as many chimpanzee read counts in Xrec1. **(B)** Reads that span the inter-specific recombination site in Xrec1 align to the appropriate locations in human chrX and chimpanzee chrX. The recombination site is a 4bp microhomology (highlighted region in close-up) that is found in both human chrX and chimpanzee chrX at the indicated coordinates.

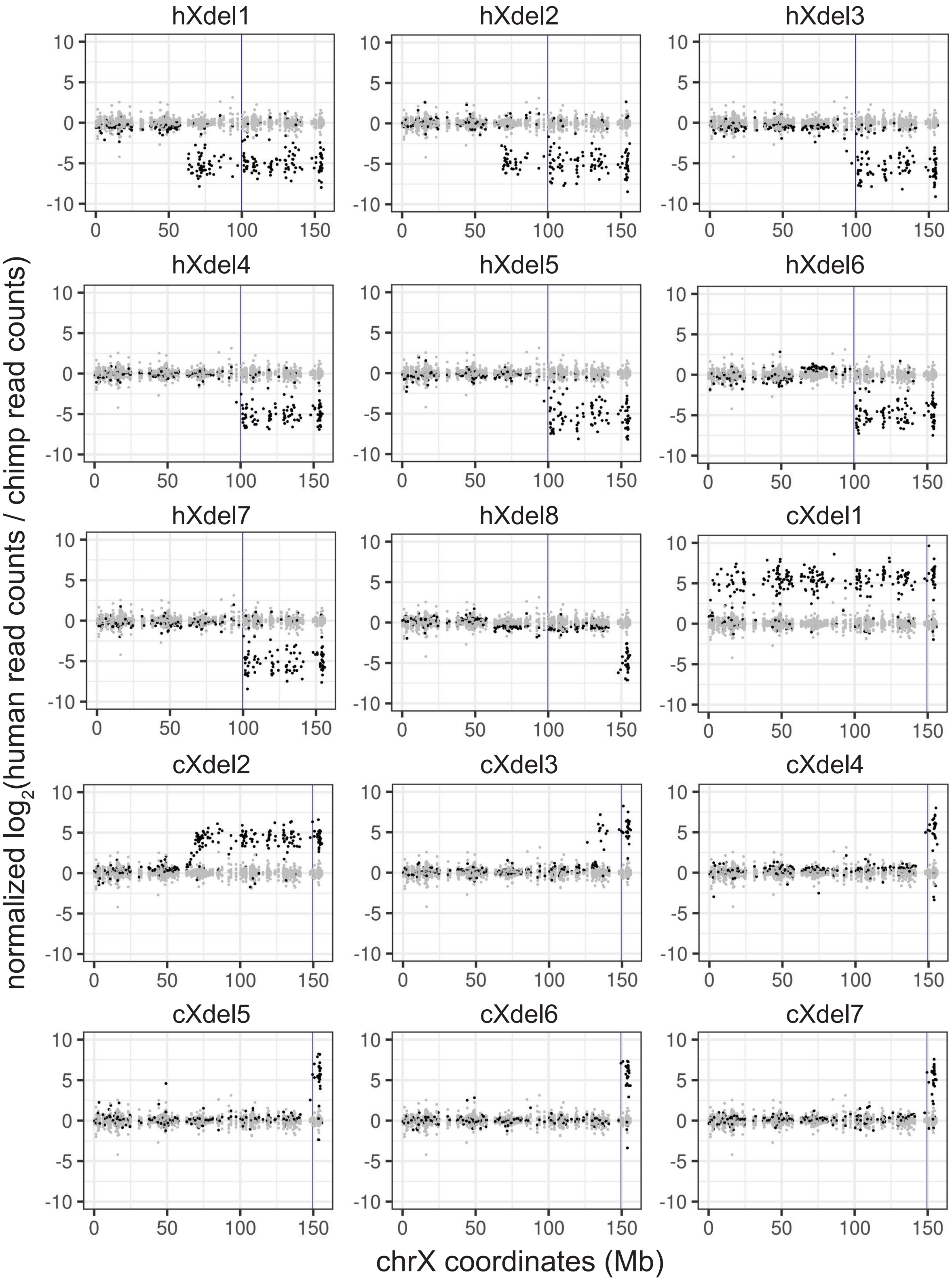

Fig. S11. RNAseq of chrX lines localizes terminal deletion breakpoints. The relative allelic expression of genes along chromosome X is plotted for each of the chrX deletion lines (Dataset S1). The y-axis is the ratio of reads that map to the human or chimpanzee allele in the deletion line (black) normalized to the ratio of reads that map to the human or chimpanzee allele in the control (non-deletion) lines (gray) (see SI Methods). Each dot represents a gene on chromosome X plotted along the x-axis at its hg38 coordinate. The vertical line is the species-specific gRNA target site used to generate each deletion line.

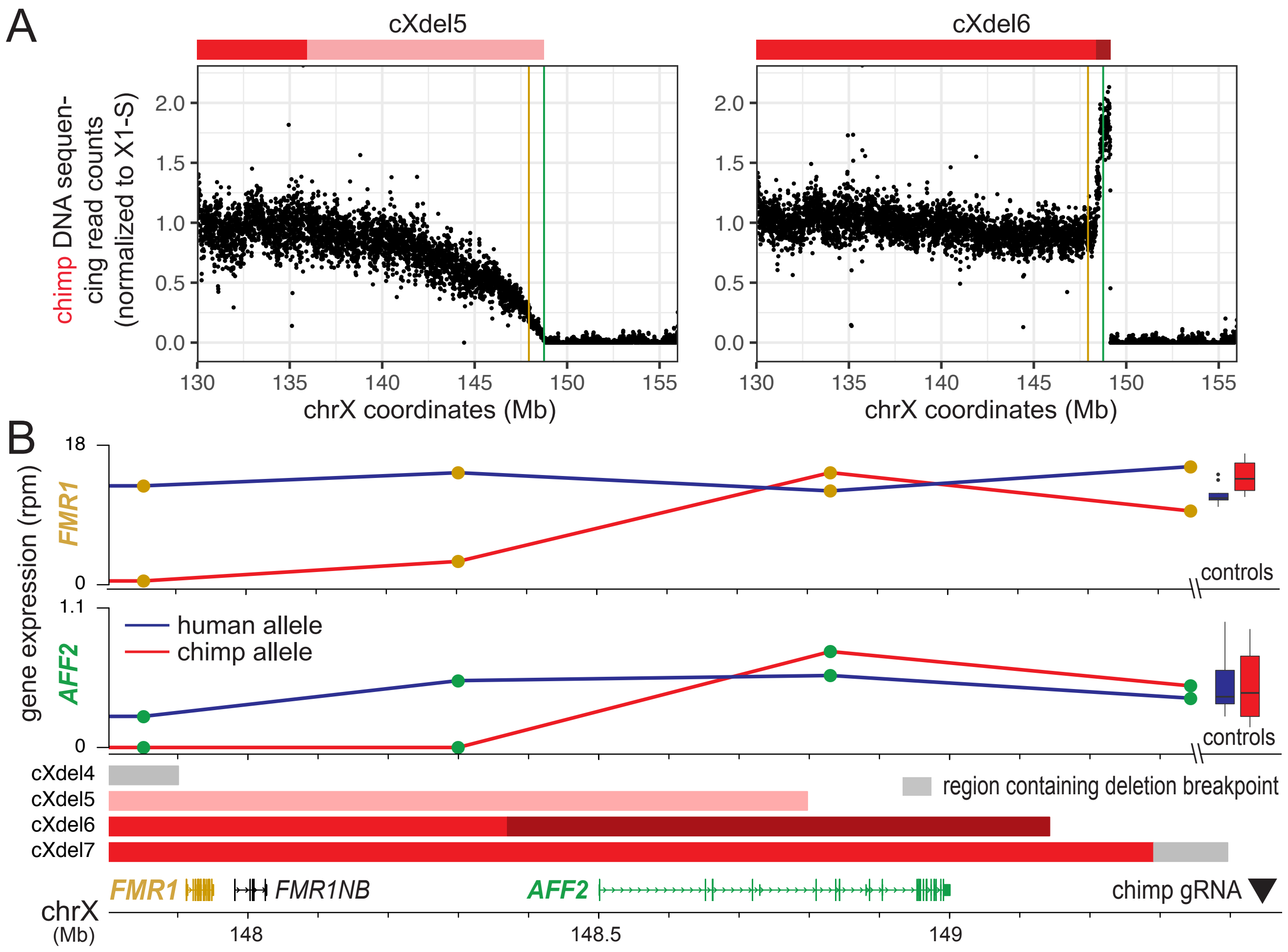

Fig. S12. Mapping chromosome breakpoints and gene expression levels in chrX deletion lines. (A) Whole-genome DNA sequencing was performed for two chimpanzee chrX deletion lines, cXdel5 and cXdel6. The ratio of the read counts that align to the chimpanzee allele for each deletion line was normalized to a control line, X1-S, and plotted along the X chromosome in hg38 coordinates (SI Methods). Colored bars above each plot indicate regions showing evidence of staggered deletions in cXdel5 (pink) or staggered insertions in cXdel6 (dark red), likely arising from a mixture of endpoints within the cell lines. Yellow line: location of *FMR1*. Green line: location of *AFF2*. **(B)** Expression of human alleles of *FMR1* or *AFF2* in the four chimpanzee chrX deletion lines is similar to control lines, as expected. Expression of the chimpanzee alleles of *FMR1* and *AFF2* is missing in cXdel4, whose terminal deletion includes both genes. Expression of chimpanzee *FMR1* is lower in cXdel5, likely corresponding to heterogeneous deletion of the gene in approximately ∼75% of cells (panel A above). Expression of chimpanzee *FMR1* and *AFF2* appears normal in cXdel6 and cXdel7, whose terminal deletions do not include these loci. Red: regions of chimpanzee chrX present in line. Pink: region in cXdel5 containing non-clonal deletions. Dark red: region in cXdel6 containing non-clonal insertions. Gray: regions containing deletion breakpoints based on PCR assays and gene expression profiling (Materials and Methods).

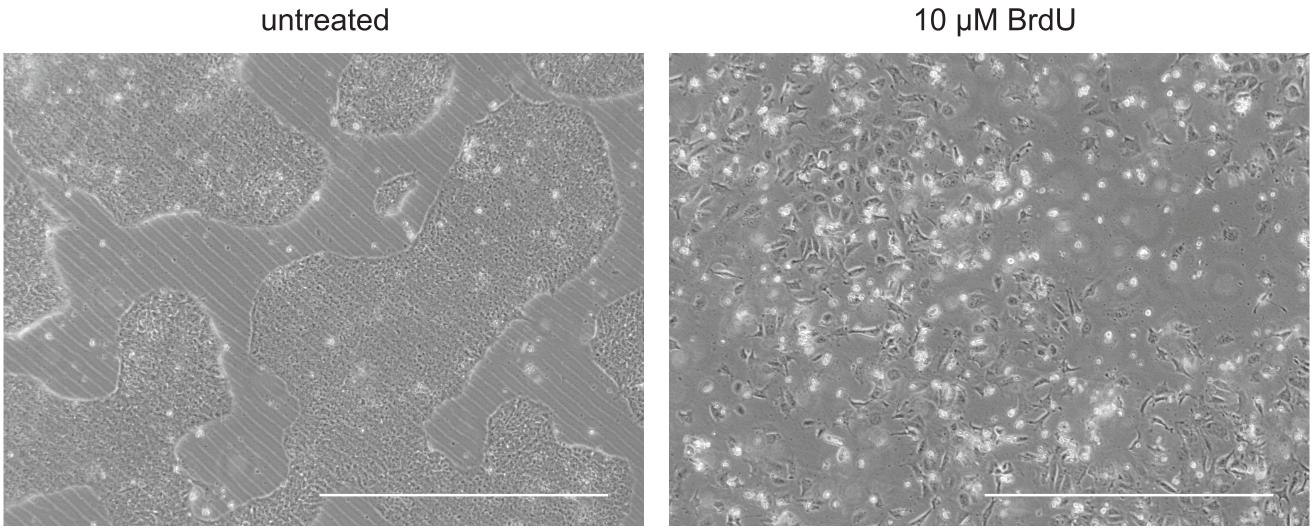

Fig. S13. BrdU induces differentiation of iPSCs. After passaging, iPSCs treated with 10μM of BrdU (right panel) are flatter and more spread out compared to untreated iPSCs (left panel). BrdU-treated cells also do not form the colonies typical of iPSCs and fail to divide, suggesting that they have terminally differentiated. Scale bars are 1mm.

SI Dataset S1 (Dataset S1)

iPSC lines used and generated in the current study.

SI Dataset S2 (Dataset S2)

Primers and gRNAs.

SI Dataset S3 (Dataset S3)

Trilineage differentiation results.

SI Dataset S4 (Dataset S4)

Differential gene expression analysis of diploid and auto-tetraploid iPSC lines.

SI Dataset S5 (Dataset S5)

Differential gene expression, allele-specific gene expression, and regulatory type (*cis/trans*) analysis between humans and chimpanzees in diploid, auto-tetraploid, and allo-tetraploid iPSC lines.

SI Dataset S6 (Dataset S6)

Gene ontology enrichments for regulatory type (*cis/trans*) categories.

SI Dataset S7 (Dataset S7)

qPCR and PCR results on chrX for sorted colonies treated with CRISPR+ML216.

SI Dataset S8 (Dataset S8)

Differential gene expression analysis of chrX deletion iPSC lines.

References

1. I Gallego Romero, et al., A panel of induced pluripotent stem cells from chimpanzees: A resource for comparative functional genomics. *eLife* **4**, e07103 (2015).
2. J Beers, et al., Passaging and colony expansion of human pluripotent stem cells by enzyme-free dissociation in chemically defined culture conditions. *Nat. Protoc.* **7**, 2029–2040 (2012).
3. KM Loh, et al., Mapping the pairwise choices leading from pluripotency to human bone, heart, and other mesoderm cell types. *Cell* **166**, 451–467 (2016).
4. B Howe, A Umrigar, F Tsien, Chromosome preparation from cultured cells. *J. Vis. Exp.*, e50203 (2014).
5. O Fedrigo, et al., A pipeline to determine RT-qPCR control genes for evolutionary studies: Application to primate gene expression across multiple tissues. *PLoS ONE* **5**, e12545 (2010).
6. C Bock, et al., Reference maps of human ES and iPS cell variation enable high-throughput characterization of pluripotent cell lines. *Cell* **144**, 439–452 (2011).
7. CE Filby, et al., Stimulation of Activin A/Nodal signaling is insufficient to induce definitive endoderm formation of cord blood-derived unrestricted somatic stem cells. *Stem Cell Res. & Ther.* **2**, 16 (2011).
8. Y Panina, A Germond, S Masui, TM Watanabe, Validation of common housekeeping genes as reference for qPCR gene expression analysis during iPS reprogramming process. *Sci. Reports* **8**, 8716 (2018).
9. YL Kuang, et al., Evaluation of commonly used ectoderm markers in iPSC trilineage differentiation. *Stem Cell Res.* **37**, 101434 (2019).
10. A Gunne-Braden, et al., *GATA3* mediates a fast, irreversible commitment to *BMP4*-driven differentiation in human embryonic stem cells. *Cell Stem Cell* **26**, 693–706.e9 (2020).
11. Y Li, et al., Generation of an induced pluripotent stem cell line SDUBMSi005-A from a patient with double primary gastric and colon carcinoma. *Stem Cell Res.* **53**, 102253 (2021).
12. M Martin, Cutadapt removes adapter sequences from high-throughput sequencing reads. *EMBnet J.* **17**, 10–12 (2011).
13. S Andrews, et al., *FastQC.* (2010) Babraham Institute.
14. A Dobin, et al., STAR: Ultrafast universal RNA-seq aligner. *Bioinformatics* **29**, 15–21 (2013).
15. Y Liao, GK Smyth, W Shi, featureCounts: An efficient general purpose program for assigning sequence reads to genomic features. *Bioinformatics* **30**, 923–930 (2014).
16. AD Yates, et al., Ensembl 2020. *Nucleic Acids Res.* **48**, D682–D688 (2020).
17. J Zhu, et al., Comparative genomics search for losses of long-established genes on the human lineage. *PLoS Comput. Biol.* **3**, e247 (2007).
18. A McKenna, et al., The Genome Analysis Toolkit: A MapReduce framework for analyzing next-generation DNA sequencing data. *Genome Res.* **20**, 1297–1303 (2010).
19. GA Van der Auwera, et al., From FastQ data to high confidence variant calls: The Genome Analysis Toolkit best practices pipeline. *Curr. Protoc. Bioinforma.* **43**, 1–11 (2013).
20. MI Love, W Huber, S Anders, Moderated estimation of fold change and dispersion for RNA-seq data with DESeq2. *Genome Biol.* **15**, 550 (2014).
21. CJ McManus, et al., Regulatory divergence in *Drosophila* revealed by mRNA-seq. *Genome Res.* **20**, 816–825 (2010).
22. X Shi, et al., *Cis*- and *trans*-regulatory divergence between progenitor species determines gene-expression novelty in *Arabidopsis* allopolyploids. *Nat. Commun.* **3**, 950 (2012).
23. HB Fraser, Improving estimates of compensatory *cis–trans* regulatory divergence. *Trends Genet.* **35**, 88 (2019).
24. G Yu, LG Wang, Y Han, QY He, clusterProfiler: An R package for comparing biological themes among gene clusters. *OMICS* **16**, 284–287 (2012).
25. DM Stults, MW Killen, AJ Pierce, The sister chromatid exchange (SCE) assay. *Methods Mol. Biol.* **1105**, 439–455 (2014).
26. JI Meier, et al., Haplotype tagging reveals parallel formation of hybrid races in two butterfly species. *Proc. Natl. Acad. Sci. USA* **118**, e2015005118 (2021).
27. A Shajii, I Numanagić, B Berger, Latent variable model for aligning barcoded short-reads improves downstream analyses. *Res. Comput. Mol. Biol.* **10812**, 280–282 (2018).
28. J Navarro Gonzalez, et al., The UCSC Genome Browser database: 2021 update. *Nucleic Acids Res.* **49**, D1046–D1057 (2021).
29. HM Amemiya, A Kundaje, AP Boyle, The ENCODE blacklist: Identification of problematic regions of the genome. *Sci. Reports* **9**, 9354 (2019).
30. G Luo, et al., Cancer predisposition caused by elevated mitotic recombination in Bloom mice. *Nat. Genet.* **26**, 424–429 (2000).
31. K Yusa, et al., Genome-wide phenotype analysis in ES cells by regulated disruption of Bloom's syndrome gene. *Nature* **429**, 896–899 (2004).
32. S Lazzarano, et al., Genetic mapping of species differences via *in vitro* crosses in mouse embryonic stem cells. *Proc. Natl. Acad. Sci. USA* **115**, 3680–3685 (2018).
33. H Wickham, *ggplot2: Elegant graphics for data analysis.* (Springer-Verlag New York), (2016).
34. B Gel, E Serra, karyoploteR: An R/Bioconductor package to plot customizable genomes displaying arbitrary data. *Bioinformatics* **33**, 3088–3090 (2017).
35. JI Wucherpfennig, CT Miller, DM Kingsley, Efficient CRISPR-Cas9 editing of major evolutionary loci in sticklebacks.

Evol. Ecol. Res. **20**, 107–132 (2019).
36. EK Brinkman, T Chen, M Amendola, B van Steensel, Easy quantitative assessment of genome editing by sequence trace decomposition. *Nucleic Acids Res.* **42**, e168–e168 (2014).
37. Y Yoshimura, A Yamanishi, T Kamitani, JS Kim, J Takeda, Generation of targeted homozygosity in the genome of human induced pluripotent stem cells. *PLoS ONE* **14**, e0225740 (2019).
38. H Li, R Durbin, Fast and accurate short read alignment with Burrows-Wheeler transform. *Bioinformatics* **25**, 1754–1760 (2009).
39. H Li, et al., The Sequence Alignment/Map format and SAMtools. *Bioinformatics* **25**, 2078–2079 (2009).
40. RM Agoglia, et al., Primate cell fusion disentangles gene regulatory divergence in neurodevelopment. *Nature* **592**, 421–427 (2021).
41. D Gokhman, et al., Gene ORGANizer: Linking genes to the organs they affect. *Nucleic Acids Res.* **45**, W138–W145 (2017).
42. L Yu, et al., Core pluripotency factors promote glycolysis of human embryonic stem cells by activating *GLUT1* enhancer. *Protein & Cell* **10**, 668–680 (2019).
43. SP Bharathan, et al., Systematic evaluation of markers used for the identification of human induced pluripotent stem cells. *Biol. Open* **6**, 100–108 (2017).
